# Complement-activating donor-specific anti-HLA antibodies in solid organ transplantation: systematic review, meta-analysis, and critical appraisal

**DOI:** 10.3389/fimmu.2023.1265796

**Published:** 2023-10-02

**Authors:** Solaf Al-Awadhi, Marc Raynaud, Kevin Louis, Antoine Bouquegneau, Jean-Luc Taupin, Olivier Aubert, Alexandre Loupy, Carmen Lefaucheur

**Affiliations:** ^1^ Université de Paris Cité, Institut National de la Santé et de la Recherche Médicale (INSERM) Unité Mixte de Recherche (UMR)-S970, Paris Cardiovascular Research Center (PARCC), Paris Translational Research Centre for Organ Transplantation, Paris, France; ^2^ Kidney Transplant Department, Saint-Louis Hospital, Assistance Publique - Hôpitaux de Paris, Paris, France; ^3^ Department of Nephrology, Dialysis and Transplantation, Centre Hospitalier Universitaire (CHU) de Liège, Liège, Belgium; ^4^ Department of Immunology and Histocompatibility, Centre Hospitalier Universitaire (CHU) Paris–GH St–Louis Lariboisière, Paris, France; ^5^ Kidney Transplant Department, Necker Hospital, Assistance Publique – Hôpitaux de Paris, Paris, France

**Keywords:** complement-activation, donor specific antibodies, anti-HLA, rejection, transplantation outcomes

## Abstract

**Introduction:**

Several studies have investigated the impact of circulating complement-activating anti-human leukocyte antigen donor-specific antibodies (anti-HLA DSAs) on organ transplant outcomes. However, a critical appraisal of these studies and a demonstration of the prognostic value of complement-activating status over anti-HLA DSA mean fluorescence intensity (MFI) level are lacking.

**Methods:**

We conducted a systematic review, meta-analysis and critical appraisal evaluating the role of complement-activating anti-HLA DSAs on allograft outcomes in different solid organ transplants. We included studies through Medline, Cochrane, Scopus, and Embase since inception of databases till May 05, 2023. We evaluated allograft loss as the primary outcome, and allograft rejection as the secondary outcome. We used the Newcastle-Ottawa Scale and funnel plots to assess risk of bias and used bias adjustment methods when appropriate. We performed multiple subgroup analyses to account for sources of heterogeneity and studied the added value of complement assays over anti-HLA DSA MFI level.

**Results:**

In total, 52 studies were included in the final meta-analysis (11,035 patients). Complement-activating anti-HLA DSAs were associated with an increased risk of allograft loss (HR 2.77; 95% CI 2.33-3.29, p<0.001; I²=46.2%), and allograft rejection (HR 4.98; 95% CI 2.96-8.36, p<0.01; I²=70.9%). These results remained significant after adjustment for potential sources of bias and across multiple subgroup analyses. After adjusting on pan-IgG anti-HLA DSA defined by the MFI levels, complement-activating anti-HLA DSAs were significantly and independently associated with an increased risk of allograft loss.

**Discussion:**

We demonstrated in this systematic review, meta-analysis and critical appraisal the significant deleterious impact and the independent prognostic value of circulating complement-activating anti-HLA DSAs on solid organ transplant risk of allograft loss and rejection.

## 1 Introduction

Antibody-mediated rejection has been identified as the main cause for allograft loss (1) and the prognostic role of circulating anti-human leukocyte antigen donor-specific antibodies (anti-HLA DSAs) has been extensively assessed across different solid organ transplants (2–5). One key characteristic of anti-HLA DSAs is their ability to undergo class-switch recombination and activate complement by fixing complement fractions. Several studies have been conducted to evaluate the impact of complement-activating anti-HLA DSAs on allograft outcomes. The reported results were heterogeneous with some studies demonstrating a strong association of complement-activating anti-HLA DSA with adverse allograft outcomes (6, 7) while others showed no or weak associations (8, 9).

As a consequence, our team previously performed a systematic review and meta-analysis to study the role of complement-activating anti-HLA DSAs on adverse allograft outcomes (10) and showed that circulating complement-activating anti-HLA DSAs increased the risk of allograft loss and rejection. However, since the publication of the review in May 2018, major studies assessing the effect of circulating complement-activating anti-HLA DSAs on allograft outcomes have been conducted (11, 12).

In addition, the quality and risk of bias of the previous and recent studies have not been evaluated and a critical appraisal remains to be performed. The Sensitization in transplantation: Assessment of Risk (STAR) working group have recently highlighted several gaps regarding whether ancillary complement-based assays (C1q, C3d, C4d) provide additional useful clinical information compared to mean fluorescence intensity (MFI) values provided by single antigen bead (SAB) pan-IgG assay (6, 8, 13, 14). Therefore, STAR working group recommends verify the role of complement binding assays *in vivo* as potential markers for adverse outcomes before recommending its use in clinical practice.

Therefore, the aim of this article was to provide a comprehensive up-to-date systematic review, meta-analysis and critical appraisal of studies testing the effect of circulating complement-activating anti-HLA DSAs on allograft outcomes and to evaluate and adjust for risk of bias.

## 2 Methods

This study was an incremental update of a systematic review and a meta-analysis (10), supplemented by a critical appraisal. The study was reported according to the Preferred Reporting Items for Systematic Reviews and Meta-Analyses (PRISMA) (15).

### 2.1 Data sources

A comprehensive search strategy was conducted on Medline, Cochrane, Scopus and Embase since inception of databases till January 31, 2018 (10). For the period between the closing date of the previous review (10) and May 05, 2023 we created a search strategy using a complementary combination of two PubMed search strategies: 1) narrow Boolean which consists of the main Medical Subject Heading (MeSH) for the population combined with the main MeSH for the intervention (see **Supplementary** for details), and 2) ranking strategy which consisted of screening all the studies listed under the “similar articles” feature on PubMed of the three largest and three newest studies included in the previous review (16). We opted for a PubMed-only database search for this period because the included articles of the previous review (10), whose search strategy was comprehensive and included multiple databases, were all indexed in PubMed (17). This search strategy was further complemented by a manual search for potential additional studies.

### 2.2 Study selection

The inclusion criteria were studies evaluating the effect of complement-activating anti-HLA DSAs on allograft loss and rejection in adult and paediatric solid organ transplant recipients. Two independent reviewers (SAA and AB) screened the titles and abstracts of the studies and any disagreement was resolved by consensus.

### 2.3 Data extraction

We collected the same data variables as the previous review: “author name, year of publication, study size, mean or median follow-up time, mean age of population, type of complement-activating anti-HLA DSA, comparison used (patients with complement-activating anti-HLA DSAs were either compared to patients without complement-activating anti-HLA DSAs, patients with non-complement activating anti-HLA DSAs detected, or a mixed group of patients without anti-HLA DSAs and with non-complement activating anti-HLA DSAs), effect sizes (HR and/or OR) and their 95% confidence intervals, potential confounding factors, and unadjusted and adjusted estimated risks of graft loss or graft rejection.” (10).

### 2.4 Critical appraisal

We used the Newcastle-Ottawa Scale (NOS) to assess the risk of bias in observational studies (18). A high NOS score (≥ 6) represents high methodological quality. Using this quality score, each study is judged on eight items which are divided into three components: selection of the study groups (up to four points), confounding variables adjustment quality (up to two points) and the outcome studied (up to three points). (see **Supplementary** for details).

Extraction of data and assessment of risk of bias was done by two independent reviewers (SAA and AB) and any disagreement was resolved by consensus.

### 2.5 Data synthesis and analysis

We performed the meta-analysis through a random-effects model with restricted maximum likelihood approach using an inverse-variance to incorporate a measure of the anticipated heterogeneity into the weight of the studies (19). The index group was complement-activating anti-HLA DSA positive patients. They were compared to either complement-activating anti-HLA DSA negative patients, anti-HLA DSA negative patients, or a mixed group of both. The pooled effect size, study weights and amount of study heterogeneity were represented by forest plots for allograft loss and rejection.

### 2.6 Statistical heterogeneity and small-study effects

We evaluated statistical heterogeneity using I² index which reflects the percentage of variability in the effect size caused by heterogeneity rather than by chance alone. An I² above 50% represented substantial heterogeneity (19).

We used a funnel plot to visually assess for the presence of small-size effects which occurs when smaller studies show different, often more pronounced effect size. We statistically assessed any asymmetry in the funnel plot with the Egger’s test (20). If this test was significant, we adjusted for small-study effects by using the precision-effect test (PET). This method provided an estimate of the effect size in a study with a hypothetical infinite sample size and thus eliminating small-study effects bias (21).

We tested for publication bias by using a contour-enhanced funnel plot (22). If a bias was observed, we adjusted by using the p-uniform* selection model which assumes that studies with statistically non-significant p-values are published with the same probability as statistically significant results (21).

### 2.7 Subgroup analyses

We performed the following subgroup analyses to address potential sources of heterogeneity in studies assessing graft loss:

#### 2.7.1 High versus low methodological quality of studies

We separately meta-analysed higher quality studies (NOS scores ≥ 6) (23) versus lower quality studies (NOS score ≤ 5).

#### 2.7.2 Comparator group used

We separately meta-analysed studies comparing complement activating anti-HLA DSA positive patients with complement activating anti-HLA DSA and studies comparing complement activating anti-HLA DSA positive patients with complement activating anti-HLA DSA negative patients and anti-HLA DSA negative patient.

#### 2.7.3 Type of organ transplanted

We separately meta-analysed studies based on the type of the transplanted organ (kidney, liver, lung, heart, pancreas and intestine). We also separately meta-analysed kidney transplants versus all other organs based on the assumption that a low number of studies are available per organ.

#### 2.7.4 Timing of antibody detection

We separately meta-analysed studies testing patients with preformed anti-HLA DSAs (defined as antibodies positive before or at the time of transplantation), *de novo* anti-HLA DSAs (defined as antibodies positive only after transplantation), or a combined group of *de novo* both.

#### 2.7.5 Type of complement-activating capacity of antibodies

We separately meta-analysed anti-HLA DSA according to their C1q-, C3d-, or C4d-, binding capacity or according to their IgG subclass.

#### 2.7.6 Thresholds for complement-activating anti-HLA DSA positivity

We separately meta-analysed studies that considered different MFI thresholds for complement-activating anti-HLA DSA positivity of 300, 500 or 1000.

### 2.8 Sensitivity analysis

We separately meta-analysed the newly identified studies since the publication of the previous review in 2018 and assessed the association of complement activating anti-HLA DSA with the risk allograft loss and allograft rejection.

### 2.9 Cumulative meta-analysis

We conducted a cumulative meta-analysis to show the change of hazard ratio of allograft loss as each study is added to the pool (24), which allowed to assess the stability of evidence i.e., whether additional studies change the overall effect of complement-binding anti-HLA DSAs on the outcome, and the sufficiency of evidence i.e., whether additional studies were needed to establish the same conclusion (25). The cumulative meta-analysis was represented on a forest-plot and the studies were arranged in a chronological order by year and month of publication.

### 2.10 Added prognostic value of complement-activating anti-HLA DSA status over anti-HLA DSA MFI level

We identified studies that showed a correlation between complement-activating anti-HLA DSA status and pan-IgG anti-HLA DSA defined by MFI levels. Then, we identified and separately meta-analysed studies that conducted multivariable analyses adjusting complement-activating anti-HLA DSA status on pan-IgG anti-HLA DSA defined by MFI levels to assess the prognostic value of complement-activating anti-HLA DSA over standard SAB pan-IgG assays.

In addition, to assess the added prognostic value of complement-activating anti-HLA DSA over EDTA treated SAB assays, we identified studies that pre-treated sera with ethylenediaminetetraacetic acid (EDTA) as means to overcome complement interference – a shortcoming of SAB assays caused by complement activation which usually results in underestimating or completely masking strong DSAs (26).

### 2.11 Added prognostic value of complement-activating anti-HLA DSA status over anti-HLA DSA class

We identified and separately meta-analysed studies that performed multivariable models adjusting complement-activating anti-HLA DSA status on DSA class to assess the independent prognostic value of complement-activating anti-HLA DSA.

The meta-analyses were conducted on R 4.1.1. All tests were two-sided, and a p-value lower than 0.05 was considered significant.

## 3 Results

### 3.1 Study identification

**Figure 1:**
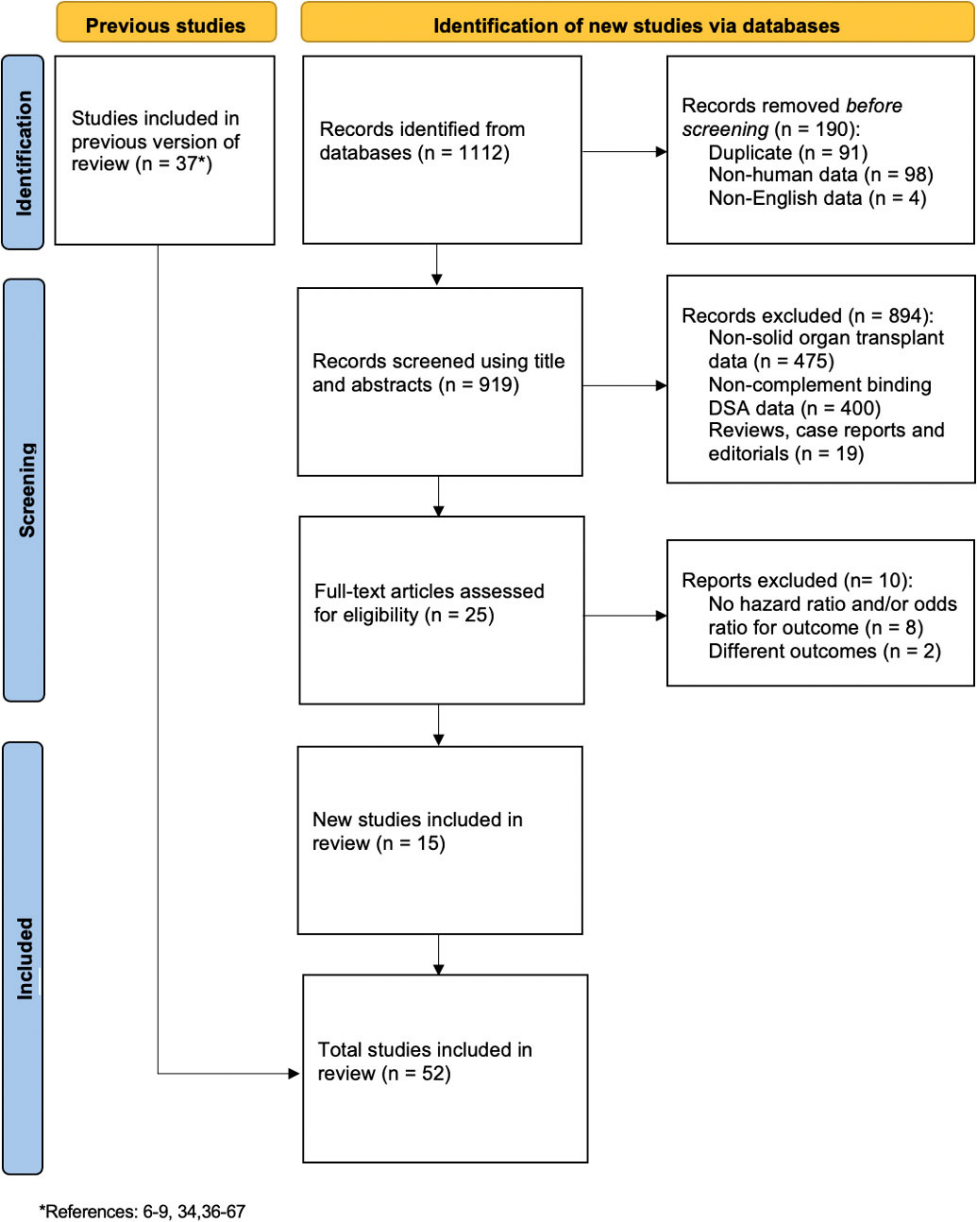
Flow chart summarizing study identification and selection process.

The search strategy identified 1,112 potential studies. After removing duplicates (n=91), studies with non-human data or not written in English (n=102), studies with non-solid organ transplant data (n=475), studies with non-complement binding anti-HLA DSAs (n=400), non-original articles (n=19), and studies with different outcomes or without hazard ratio/odds ratio (n=10), 15 new studies were identified, corresponding to 3,099 patients (**Figure 1**). The previous review (10) included 37 studies, therefore, in this incremental update, 52 studies in total were included in the final meta-analysis, corresponding to 11,035 patients. A descriptive summary of all the included studies is shown in **Table 1**.

**Table 1:** Description of the 52 included studies.

First author (date of publication)	Population	Study type	Period of inclusion	Sample size	Type of C’ anti-HLA DSA	MFI threshold for DSA detection	MFI threshold for complement positivity	EDTA pre-treatment	Outcome	Effect size (95% CI)
**Wahrmann et al. (2009)** (27)	Retrospective, single-centre analysis of consecutive adult renal transplants selected based on the presence of pretransplant DSAs	Cohort	2001-2002	338	C4d	Not reported	Not reported	Not reported	Graft loss Rejection	2.40 (0.90-6.00) 10.10 (3.20-31.00)
**Hönger et al. (2010)** (28)	Retrospective, single-centre analysis of consecutive adult renal transplant recipients with low levels of pretransplant DSAs	Cohort	1999-2004	64	C4d	>500	Not reported	No	Rejection	0.93 (0.25-3.44)
**Sutherland et al. (2011)** (29)	Retrospective, single-centre analysis of paediatric renal transplant recipients without DSAs at the time of transplantation	Cohort	2000-2008	35	C1q	>1000	>450	No	Graft loss	5.80 (1.40-22.90)
**Hönger et al. (2011)** (30)	Retrospective, single-centre analysis of adult renal transplant recipients with high levels of DSAs pre transplant; recipients who developed AMR within 6 months	Cohort	1999-2008	71	IgG3	>500	Not reported	No	Rejection	0.43 (0.17-1.12)
**Smith et al. (2011)** (31)	Retrospective, single-centre analysis of living heart transplant recipients after 1 year of transplantation without DSAs pre-transplant	Cohort	1995-2004	243	C4d	>1000	Not reported	No	Graft loss	3.02 (1.11-8.23)
**Kaneku et al. (2012)** (32)	Retrospective (2-centre) analysis of adult liver transplant recipients with liver biopsies showing chronic rejection and DSA analysis at the same time	Case-control	NC	39	IgG3	>5000	Not reported	No	Graft loss	3.35 (1.39-8.05)
**Bartel et al. (2013)** (33)	Retrospective, single-centre analysis of 68 desensitized renal recipients who had been subjected to peri-transplant desensitization	Cohort	1999-2008	68	C4d	>500	Not reported	No	Rejection	10.10 (1.60-64.20)
**Lawrence et al. (2013)** (34)	Retrospective, single-centre study of consecutive renal transplant recipients	Cohort	2005-2010	52	C4d	>300	Not reported	No	Rejection	8.90 (1.20-65.86)
**Crespo et al. (2013)** (8)	Retrospective (2-left) analysis of renal transplant patients with pretransplant DSAs	Cohort	2006-2011	355	C1q	>2000	>500	Yes	Graft loss Rejection	0.83 (0.17-4.14) 1.44 (0.23-9.11)
**Loupy et al. (2013)** (6)	Consecutive adult patients in a retrospective (2-left) analysis; unselected global population with DSA detection before or after renal transplantation	Cohort	2004-2010	1,016	C1q	>500	>500	No	Graft loss	4.78 (2.69-8.49)
**Freitas et al. (2013)** (35)	Retrospective, single-centre analysis of renal transplant recipients selected on the basis of DSA detection during follow-up	Cohort	1999-2012	203	IgG3	>1000	>500	No	Graft loss	3.50 (1.30-9.50)
**Arnold et al. (2014)** (36)	Retrospective, single-centre analysis of renal transplant recipients without DSAs pre transplant and screened for *de novo* DSAs	Cohort	1997-2007	274	IgG3	>1000	>500	No	Graft loss	4.81 (1.65-14.03)
**Smith et al. (2014)** (37)	Retrospective, single-centre analysis of lung transplant recipients with pretransplant DSA detection	Cohort	1991-2003	63	C4d	500	Not reported	No	Graft loss	6.43 (2.96-13.97)
**Everly et al. (2014)** (38)	Retrospective, single-centre analysis of primary renal transplant recipients without pretransplant DSA detection	Cohort	1999-2006	179	IgG3	>1000	>1000	No	Graft loss	2.48 (1.02-6.04)
**O’Leary et al. (2015)** (39)	Retrospective, single-centre analysis of ƒconsecutive patients with 1-year survival post liver transplantation; one group analysed pretransplant DSA effects, and another group analysed the impact of *de novo* DSAs	Cohort	2000-2009	1,270	C1q IgG3	>5000	>500	No	Graft loss Graft loss	1.90 (1.62-3.45) 2.40 (1.82-5.75)
**Wozniak et al. (2015)** (40)	Retrospective, single-centre analysis of paediatric liver transplant patients who were either nontolerant, tolerant, or stable	Cohort	NC	50	C1q	>1000	>1000	No	Rejection	4.30 (1.10-16.40)
**Khovanova et al. (2015)** (41)	Retrospective, single-centre analysis of HLA-incompatible desensitized renal transplant patients	Cohort	2003-2012	80	IgG3	>1000	Not reported	No	Graft loss	2.09 (0.30-14.60)
**Sicard et al. (2015)** (14)	Retrospective analysis of consecutive (2-left) adult renal transplant patients who developed AMR	Cohort	2004-2012	69	C3d C1q	>500	Not reported	No	Graft loss Graft loss	2.80 (1.12-6.95) 1.98 (0.95-4.14)
**Thammanichanond et al.** **(2016)** (42)	Retrospective, single-centre cohort study of patients with pre-transplant DSAs	Cohort	2009-2013	48	C1q	>1000	>500	No	Rejection	2.20 (0.61-7.85)
**Comoli et al. (2016)** (43)	Retrospective analysis of consecutive paediatric recipients; single centre; first kidney transplant without any HLA antibodies in sera or at the time of transplantation	Cohort	2002-2013	114	C3d C1q C3d C1q	>1000	>500	No	Rejection RejectionGraft loss Graft loss	6.91 (2.78-17.18) 13.54 (4.95-36.99) 27.80 (5.61-137.72) 11.09 (2.25-54.64)
**Yamamoto et al. (2016)** (44)	Retrospective analysis of renal transplant patients with *de novo* DSAs and surveillance biopsies	Cohort	2009-2013	43	C1q	>1000	>1000	No	Rejection	2.60 (0.12-53.90)
**Calp-Inal et al. (2016)** (7)	Retrospective analysis; single centre; consecutive renal transplant patients: Group 1 without pretransplant DSAs and Group 2 with a mix of pre-existing and *de novo* DSAs	Cohort	2009-2012	284	C1q	>1000	>500	No	Graft loss	4.30 (1.10-16.50)
**Malheiro et al. (2016)** (45)	Retrospective, single-centre analysis of kidney transplant patients with DSAs pre transplant	Cohort	2007-2012	60	C1q	>1000	>500	Yes	Rejection	16.80 (3.18-88.85)
**Visentin et al. (2016)** (46)	Retrospective, single-centre analysis of lung transplant patients with biopsy (with demonstration of rejection) and serum available	Cohort	1999-2014	53	C1q	>500	>500	No	Graft loss	1.65 (0.68-3.97)
**Kauke et al. (2016)** (47)	Retrospective, single-centre analysis of patients selected based on renal biopsy-proven rejection during graft dysfunction or viremia with polyomavirus BK	Cohort	2005-2011	611	C3d C1q	1000	Not reported	No	Graft loss Rejection	3.77 (1.40-10.16) 4.52 (1.89-10.37)
**Bamoulid et al. (2016)** (48)	Retrospective, single-centre analysis of renal transplant consecutive patients without DSAs pre transplant	Cohort	2007-2014	59	C1q	>1000	>300	No	Rejection Graft loss	2.27 (1.05-4.91) 6.78 (0.86-53.50)
**Fichtner et al. (2016)** (49)	Retrospective, single-centre analysis of prospectively screened renal transplant paediatric patients, non-presensitised	Cohort	1999-2010	62	C1q	>500	>300	No	Graft loss	6.35 (1.33-30.40)
**Guidicelli et al. (2016)** (50)	Retrospective, single-centre analysis of consecutive non-sensitized kidney transplant patients	Cohort	1998-2005	346	C1q	>500	Not reported	Yes	Graft loss	2.99 (0.94-10.27)
**Lefaucheur et al. (2016)** (51)	Retrospective analysis of consecutive patients (2-left); renal transplant patients were unselected	Cohort	2008-2010	125	IgG3 C1q	>500	>500	No	Graft loss Graft loss	4.80 (1.70-13.30) 3.60 (1.10-11.70)
**Viglietti et al. (2017)** (52)	Retrospective analysis of consecutive patients (2-left); renal transplant recipients were unselected	Cohort	2008-2011	851	IgG3 C1q	>1000	>500	No	Graft loss Graft loss	4.25 (1.88-9.61) 3.60 (1.71-7.59)
**Wiebe et al. (2017)** (53)	Retrospective analysis of consecutive adult and paediatric renal transplant patients, single centre; patients without pretransplant sensitization	Cohort	1999-2012	70	C1q	>300	>300	Yes	Graft loss	1.06 (0.50-2.40)
**Moktefi et al. (2017)** (9)	Retrospective analysis (2-left) of patients selected based on the development of acute renal AMR and the presence of DSAs	Cohort	2005-2012	48	C1q	>500	>500	No	Graft loss	0.79 (0.25-2.44)
**Sicard et al. (2017)** (54)	Retrospective analysis of consecutive adult renal transplant patients (2-left) with unselected patients	Cohort	2004-2012	52	C3d	500	>500	No	Graft loss	3.71 (1.27-10.80)
**Das et al. (2017)** (55)	Retrospective, single-centre analysis of paediatric heart transplant without DSAs pre transplantation and at the time of transplantation	Cohort	2005-2014	127	C1q	>1000	>500	No	Graft loss	3.20 (1.34-7.86)
**Couchonnal et al. (2017)** (56)	Retrospective analysis; single-centre analysis of consecutive paediatric liver transplant selected on the presence of DSAs during follow-up	Cohort	1990-2014	100	C3d	>500	>500	No	Graft loss	4.12 (0.95-17.89)
**Bailly et al. (2017)** (57)	Retrospective analysis of multi-centre, prospective, randomized, double-blind, placebo-controlled, parallel-group trials; patients selected on the basis of renal AMR development and DSA detection; patients treated either with standard of care (PP plus IVIg) or rituximab plus standard of care	Cohort	2008-2011	25	C1q	>500	>500	Yes	Graft loss	3.70 (0.80-17.00)
**Molina et al. (2017)** (58)	Retrospective analysis; single-centre analysis of consecutive adult kidney transplant patients selected on pretransplant DSA detection	Cohort	1995-2009	389	C1q	>1000	>500	No	Graft loss	4.01 (2.33-6.92)
**Lan et al. (2018)** (11)	Retrospective multi-centre analysis of adult kidney transplant patients selected on the presence of preformed DSA	Cohort	Before 2005	896	C3d	>500	>500	No	Graft loss	1.04 (0.37-2.94)
**Courant et al. (2018)** (12)	Retrospective single-centre analysis of adult kidney transplant patients selected on pre-transplant DSA detection	Cohort	2004-2013	192	C1q C3d	>500	>300	Yes	Graft loss Graft loss	1.74 (0.94-3.21) 1.01 (0.51-1.98)
**Brugière et al. (2018)** (59)	Retrospective, three-centre analysis of consecutive adult lung transplant patients selected on the presence of DSA during follow-up	Cohort	2009-2012	168	C1q	>500	>300	Yes	Graft loss	2.98 (1.33-6.66)
**Kamburova et al. (2018)** (60)	Retrospective multi-centre analysis of adult kidney transplant patients selected on the presence of preformed DSA	Cohort	1995-2005	567	C3d	>750	Not reported	No	Graft loss	1.02 (0.70-1.48)
**Viglietti et al. (2018)** (61)	Retrospective two-centre analysis of consecutive adult kidney transplant patients selected on the presence of DSA during follow-up	Cohort	2008-2011	139	C1q	>1000	Not reported	No	Graft loss	2.57 (1.29-5.12)
**Lee H et al. (2018)** (62)	Retrospective single-centre analysis of adult kidney transplant patients selected on the presence of *de novo* DSA	Cohort	1988-2016	161	C1q C3d C1q C3d	>1000	>1000	Yes	Graft loss Graft loss Rejection Rejection	2.90 (1.43-5.58) 2.82 (1.46-5.43) 18.5 (5.90-58.10) 8.10 (3.00-21.60)
**Malheiro et al. (2018)** (63)	Retrospective single-centre analysis of consecutive adult kidney transplant patients selected on the presence of DSA during follow-up	Cohort	2008-2015	56	C1q	>1000	>500	No	Graft loss	3.94 (1.53-10.18)
**Schinstock et al. (2018)** (64)	Retrospective multi-centre analysis of adult solitary kidney transplant patients selected on the presence of DSA during follow-up	Cohort	1998-2015	113	C1q IgG3	>1000	>1000	No	Graft loss Graft loss	5.90 (2.30-15.60) 3.80 (1.50-9.30)
**Lee DR et al. (2018)** (13)	Retrospective single-left analysis of adult solitary kidney transplant patients selected on the presence of DSA during follow-up	Cohort	2013-2016	220	C3d	>500	>500	No	Graft loss	3.02 (1.52-12.12)
**Babu et al.** (2020) (65)	Retrospective multi-left analysis of adult solitary kidney transplant patients selected on the presence of DSA during follow-up	Cohort	2005-2015	139	C3d	Not reported	Not reported	No	Graft loss	4.56 (1.46-14.4)
**Vargas et al. (2020)** (66)	Retrospective single-left analysis of adult solitary kidney transplant patients selected on the presence of DSA during follow-up	Cohort	2003-2014	86	C1q	>1000	Not reported	No	Graft loss	1.09 (0.42-2.78)
**Zhang et al. (2018)** (67)	Retrospective single-centre analysis of paediatric heart transplant patients selected on the presence of DSA during follow-up	Cohort	2010-2013	176	C3d	>1000	>1000	No	Rejection	33.0 (8.10-138)
**Cioni et al. (2019)** (68)	Retrospective single-centre analysis of consecutive adult kidney transplant patients selected on the presence of *de novo* DSA	Cohort	2002-2014	69	C1q C3d	>1000	>500	No	Rejection Rejection	0.80 (0.20-3.10) 10.10 (1.50-68.30)
**Hayde et al. (2020)** (69)	Retrospective single-centre analysis of paediatric kidney transplant patients selected on the presence of *de novo* DSA	Cohort	2009-2016	48	C1q	>1000	>500	No	Rejection	10.10 (2.00-51.80)
**Pernin et al. (2020)** (70)	Retrospective two-centre analysis of adult kidney transplant patients selected on the presence of *de novo* DSA	Cohort	2014-2018	69	IgG3	>1000	Not reported	Yes	Rejection	11.15 (2.24-55.37)

Effect sizes refer to HR for allograft loss and OR for rejection appearance.

C’, complement; C1q, complement component 1q; CI, confidence interval; DSA, donor-specific antibody; HR, hazard ratio; IgG3, immunoglobulin G3; OR, odds ratio.

### 3.2 Study characteristics

In total, 31 (59.6%) studies originated from Europe, 13 (25.0%) from North America, 5 (9.6%) from the United Kingdom, and 3 (5.8%) from Asia. The majority of the patients were kidney recipients (n=8,746; 79.3%), followed by liver recipients (n=1,459; 13.2%), heart recipients (n=546; 4.9%), and lung recipients (n=284; 2.6%). No pancreas or intestine recipients were identified. Complement-activating anti-HLA DSAs were classified by their capacity to bind C1q (28 studies; 53.8%), C3d (12 studies; 23%), or C4d (6 studies; 11.5%) or by their IgG subclass composition (10 studies; 19%).

The median NOS score was 6 (IQR 3-9) with 1.5%, 13%, 15%, 30%, 28%, 13%, and 3% of studies having a NOS score of 3, 4, 5, 6, 7, 8, and 9, respectively. Details on the NOS scoring results are available in **Supplementary Table 1**.

### 3.3 Outcomes

#### 3.3.1 Risk for allograft loss

**Figure 2:**
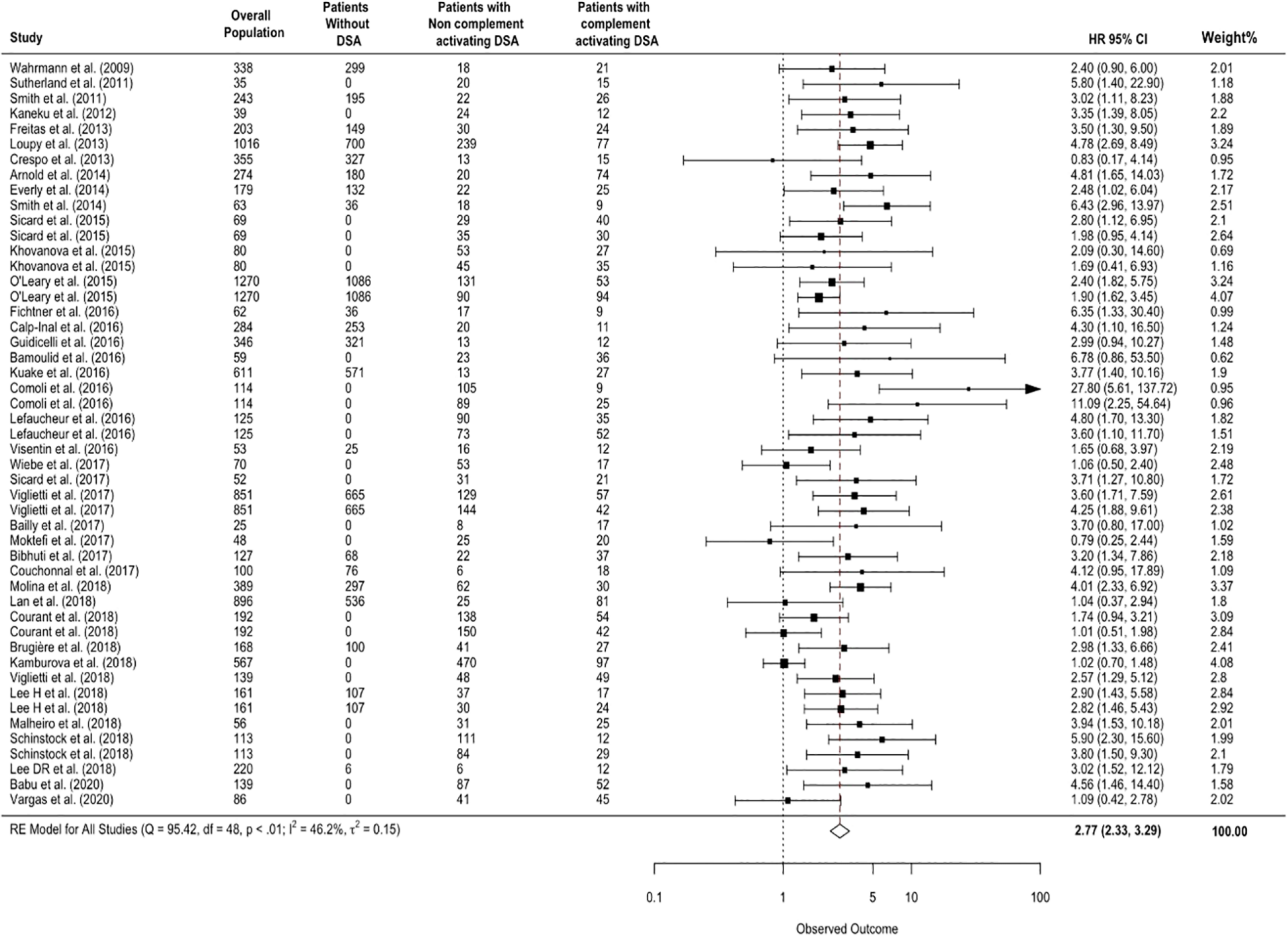
Association between circulating complement-activating anti-HLA DSAs and the risk of allograft loss. The figure shows the forest plot of the association between complement-activating anti-HLA DSAs and the risk of allograft loss for each complement binding study and overall (n = 49). Studies are listed by date of publication. Number of patients are listed in the 4 cohort columns. The black square-shaped boxes represent the HR for each individual study. The size of these boxes represents the weight of the study, and lines represent the 95% CI for individual studies. The diamond at the bottom represents the pooled HR. The number of patients in the overall population does not correspond to the sum of the different groups for the studies of Kaneku et al. (32) (3 patients), Sicard et al. (14) (4 patients), and Moktefi et al. (9) (3 patients) either because the data for these patients were missing or because they were not involved in the analysis. CI, confidence interval; DSA, donor-specific antibody; HLA, human leukocyte antigen; HR, hazard ratio.

Patients with complement-activating anti-HLA DSAs had a 2.77-fold increase in risk for allograft loss (95% CI 2.33-3.29, p<0.001; I²=46.2%) compared to patients without complement-activating anti-HLA DSA, patients without anti-HLA DSAs, and a mixed group of both (**Figure 2**).

#### 3.3.2 Risk of allograft rejection

**Figure 3:**
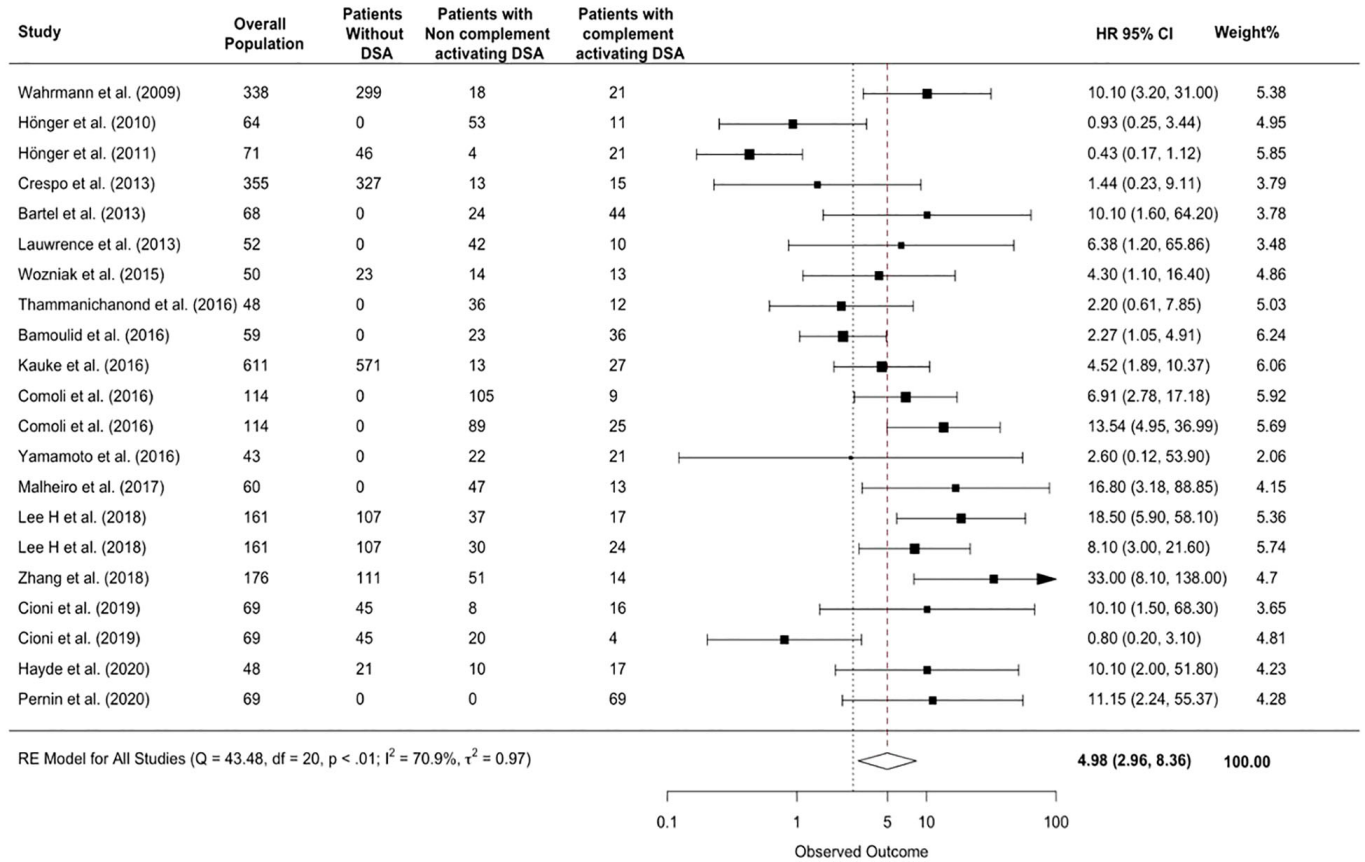
Association between complement-activating anti-HLA DSAs and the risk of rejection. The figure shows the forest plot of the association between complement activating anti-HLA DSAs and the risk of rejection for each study and overall (n = 17). Studies are listed by date of publication. The black square-shaped boxes represent the HR for each individual study. The black square-shaped boxes represent the HR for each individual study. The size of these boxes represents the weight of the study, and lines represent the 95% CI for individual studies. The diamond at the bottom represents the overall HR. CI, confidence interval; DSA, donor-specific antibody; HLA, human leukocyte antigen; HR, hazard ratio.

Patients with complement-activating anti-HLA DSAs had a 4.98-fold increase in risk of allograft rejection (95% CI 2.96-8.36, p<0.001; I²=70.9%) compared to patients without complement-activating anti-HLA DSA, patients without anti-HLA DSAs, and a mixed group of both (**Figure 3**).

### 3.4 Small-study effects

**Figure 4:**
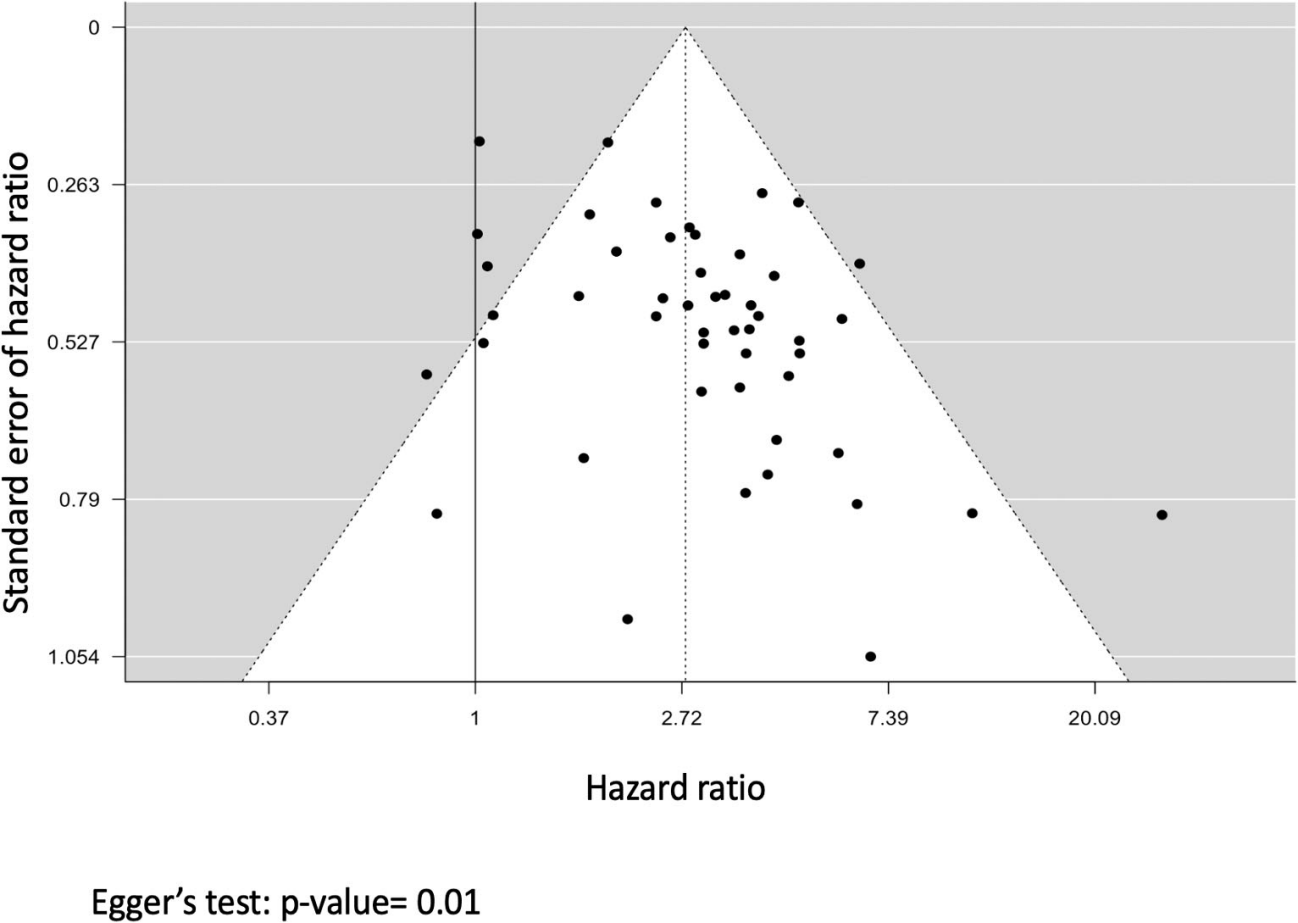
Funnel plot representing the analysis for small-study effects. Each black dot represents a study; the x-axis represents the study effect size (hazard ratio), and the y-axis represents the standard error of the hazard ratio. The dashed vertical line represents the overall risk estimate and the black line represents the no intervention effect.

Visually, the funnel plot presented in **Figure 4** showed an asymmetry which was confirmed by Egger’s test (p=0.01) indicating the presence of small-study effects. When adjusting using the PET method, the hazard ratio remains significant (HR=1.5, p<0.001) indicating that in a hypothetical infinite sample size, complement-binding anti-HLA DSAs would still increase the risk for allograft loss (**Supplementary Figure 3**).

**Figure 5:**
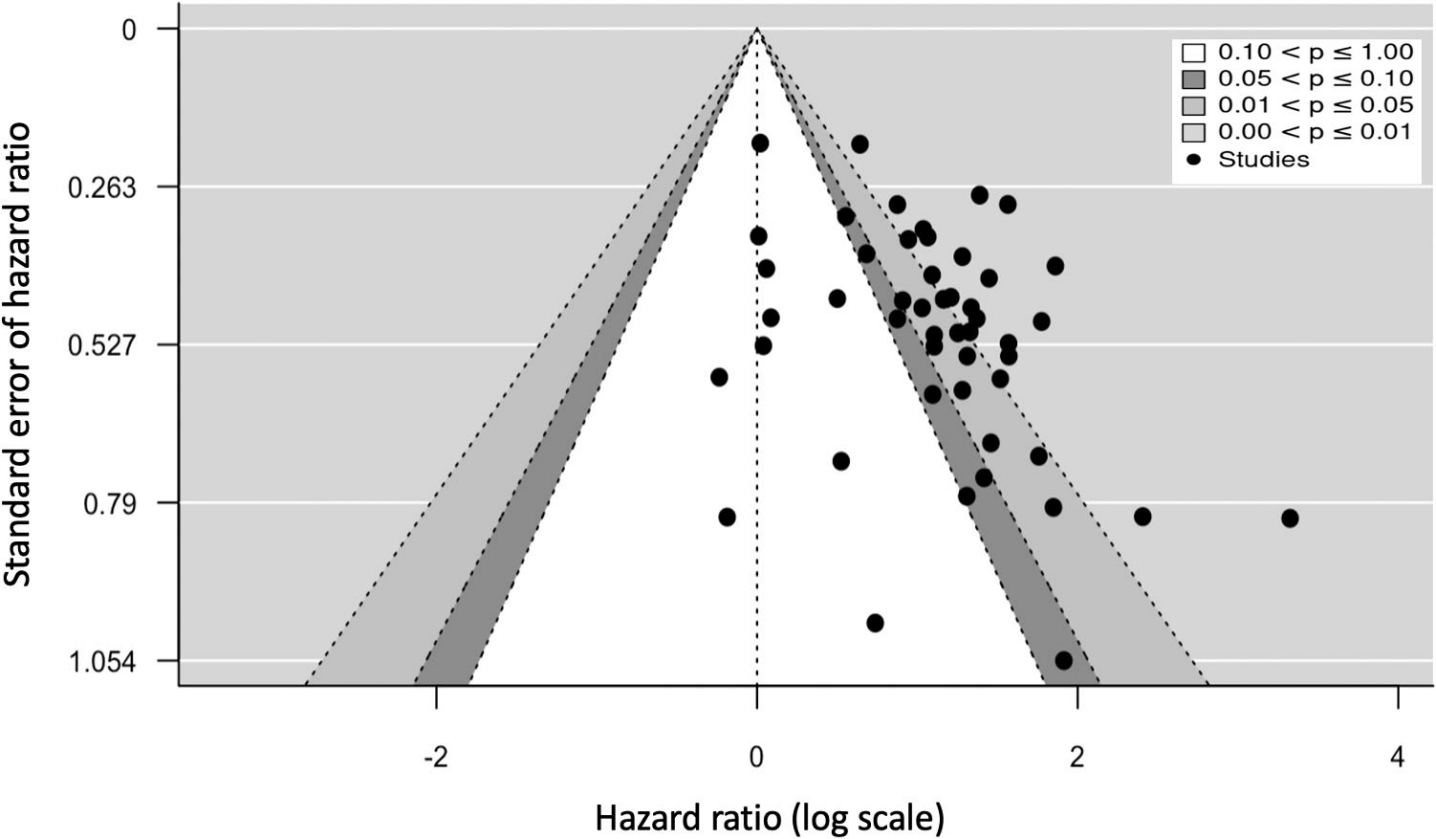
Contour-enhanced funnel plot representing the analysis for publication bias according to the statistical significance of studies. Each black dot represents a study; the x-axis represents the study effect size (hazard ratio), and the y-axis represents the standard error of the hazard ratio.

Publication bias, as a potential cause for small-study effects, was assessed using the contour-enhanced funnel plot presented in **Figure 5** which showed that more studies lie in the statistically significant side of the graph. We adjusted for this bias by using the p-uniform* selection model which yielded a hazard ratio of 2.46 (p=0.01) indicating that taking into account studies with non-significant p-values, complement-binding anti-HLA DSAs would still increase the risk of allograft loss.

### 3.5 Subgroup analysis

**Table 2** summarizes the effect sizes for each subgroup.

**Table 2:** Effect sizes related to the different subgroup analyses.

Subgroup analyses for allograft survival		Effect size	95% CI	I^2^, p-value
**Effect of complement-activating anti-HLA DSAs in studies with high or low methodological quality**	High-methodological quality studies NOS ≥6	2.79	2.33-3.35	45.7%, p<0.001
	Low-methodological quality studies NOS ≤ 5	2.46	1.28-4.70	60.5%, p<0.001
**Effect of complement-activating anti-HLA DSAs in studies with different comparators used**	Studies comparing index group and patients with non-complement-activating anti-HLA DSAs	2.56	1.99-3.30	54.2%, p< 0.001
	Studies comparing index group and patients with non-complement-activating anti-HLA DSAs and without anti-HLA DSAs	3.58	2.70-4.74	4.1%, p<0.001
**Effect of complement-activating anti-HLA DSAs according to the type of solid organ transplant**	Kidney transplantation studies only	2.77	2.25-3.41	49.2%, p< 0.001
	Heart, lung, and liver transplantation studies	2.74	2.03-3.69	29.2%, p<0.001
**Effect of complement-activating anti-HLA DSAs according to the timing of antibody detection**	Pre-existing DSAs	2.56	1.99-3.30	56.2%, p< 0.001
	Pre-existing and *de novo* DSAs	2.59	2.05-3.26	34.3%, p<0.001
	*De novo* DSAs	3.53	2.63-4.74	26.0%, p<0.001
**Effect of complement-activating anti-HLA DSAs according to the type of test used for detecting complement-activating antibodies**	C1q	2.72	2.19-3.38	35.8%, p= 0.001
	C4d	3.81	2.02-7.2	33.0%, p= 0.001
	C3d	2.50	1.48-4.25	73.1%, p< 0.001
	IgG3	3.17	2.37-4.24	0.00%, p<0.001
**Effect of complement-activating anti-HLA DSAs according to the MFI thresholds for complement-activating anti-HLA DSA positivity**	300	2.74	1.91-3.93	36.7%, p<0.001
	500	2.29	2.29-3.82	47.8%, p<0.001
	1000	2.37	1.7-3.29	49.1%, p<0.001

Effect sizes refer to HR for graft survival and OR for rejection appearance. The Index group refers to patients with complement-activating anti-HLA DSAs.

DSA, donor-specific antibody; C1q, complement component 1q; CI, confidence interval; HR, hazard ratio; HLA, human leukocyte antigen; I^2^, heterogeneity; IgG3, immunoglobulin G3; MFI, MFI, Mean fluorescent intensity, NOS, Newcastle-Ottawa scale; OR, odds ratio.

#### 3.5.1 Effect of complement-activating anti-HLA DSAs in high methodological quality studies

Analysis done on high methodological quality studies (NOS≥6) showed a significantly increased risk of allograft loss in complement activating anti-HLA DSAs positive patients with a pooled HR of 2.79 (95% CI 2.33-3.35, p<0.001, I^2 ^= 45.7%). Studies with lower methodological quality (NOS ≤ 5) also showed an increased risk of allograft loss with a HR of 2.46 (CI 1.28-4.70, p<0.001) however, as expected, the heterogeneity level between the lower methodological quality studies was higher (I^2 ^= 60.5%).

#### 3.5.2 Effect of the complement-activating anti-HLA DSAs using different comparators

The association between complement-activating anti-HLA DSAs and risk of allograft loss remained significant using different comparator groups. When comparing complement-activating anti-HLA DSAs positive patients to complement-activating anti-HLA DSAs negative patients, the pooled HR was 2.56 (95% CI 1.99-3.30, p<0.001, I^2 ^= 54.2%). When comparing, complement- activating anti-HLA DSA positive patients to a mixed group of complement- activating anti-HLA DSA negative patients and anti-HLA DSA negative patients, the pooled HR was 3.58 (95% CI 2.70-4.74, p<0.001; I^2 ^= 4.1%).

#### 3.5.3 Effect of complement-activating anti-HLA DSAs according to the type of organ transplantation

Analysis done on kidney allograft recipients versus all other solid organ allograft recipients showed a significant increased risk of allograft loss with HRs of 2.77 (CI 2.25-3.41, p<0.001; I^2 ^= 49.2%) and 2.74 (CI 2.03-3.69, p<0.001; I^2 ^= 29.2%) respectively. Analysis specific to other organs showed an increased risk for allograft loss, however, the results were not statistically significant due to the low number of studies found (**Supplementary Table 2**).

#### 3.5.4 Effect of complement-activating anti-HLA DSAs according to the timing of antibody detection

Analysis according to the time of antibody detection all showed significant associations with the highest HR of 3.53 for *de novo* DSAs (CI 2.63-4.74, p<0.001; I^2 ^= 26%).

#### 3.5.5 Analysis according to the type of complement-activating antibodies

Analysis across the different types of complement-activating antibodies showed significant overall effect on allograft loss. The following groups were assessed: (i) C1q-binding capacity (HR 2.72; 95% CI 2.19-3.38, P<0.001; I^2 ^= 35.8%), (ii) C4d-binding capacity (HR 3.81; 95% CI 2.02-7.20, p<0.001; I^2 ^= 33%), (iii) C3d-binding capacity (HR 2.50; 95% CI 1.48-4.25, p<0.001; I^2 ^= 73.1%), (iv) IgG3 subclass (HR 3.17; 95% CI 2.37-4.24, p<0.001; I^2 ^= 0.0%).

#### 3.5.6 Analysis according to MFI thresholds for complement-activating anti-HLA DSA positivity

6 (15.0%) studies used 300 as an MFI threshold for complement-activating anti-HLA DSA positivity, 1 (2.5%) study used 450, 21 (52.5%) studies used 500, 2 (5.0%) studies used 1000, and 11 (27.5%) studies did not provide the threshold value. The risk of allograft loss remained significantly increased at all complement-activating anti-HLA DSA positivity thresholds: (i) MFI 300 (HR 2.74; 95% CI 1.91-3.93, p<0.001; I^2 ^= 36.7%), (ii) MFI 500 (HR 2.96; 95% CI 2.29-3.82, p<0.001; I^2 ^= 47.8%), (iii) MFI 1000 (HR 2.37; 95% CI 1.7-3.29, p<0.001; I^2 ^= 49.1%).

### 3.6 Sensitivity analysis

The separate meta-analysis of the 15 newly identified studies since the publication of the previous review in 2018 showed that patients with complement-activating anti-HLA DSAs had a 2.21-fold increase in risk for allograft loss (95% Cl 1.61-3.04; p<0.001; I^2 ^= 58.8) (**Supplementary Figure 1**) and a 8.87-fold increase in risk for allograft rejection (95% CI 3.64-21.6; p<0.001; I^2 ^= 65.3%) compared to patients without complement-activating anti-HLA DSA, patients without anti-HLA DSAs, and a mixed group of both (**Supplementary Figure 2**).

### 3.7 Cumulative meta-analysis

The cumulative meta-analysis showed the effect of adding new studies in a chronological order on the overall effect size (**Supplementary Figure 4**). Starting at the second study in 2011 till the end of analysis, there is a consistent and statistically significant risk of allograft loss.

The cumulative meta-analysis demonstrated that adding new studies: i) narrowed the confidence intervals of the overall effect size, ii) reduced the already statistically significant p-values, iii) converged the overall effect size of complement- activating antibodies on allograft loss.

### 3.8 Added prognostic value of complement-activating anti-HLA DSA status over anti-HLA DSA MFI level on allograft loss

**Figure 6:**
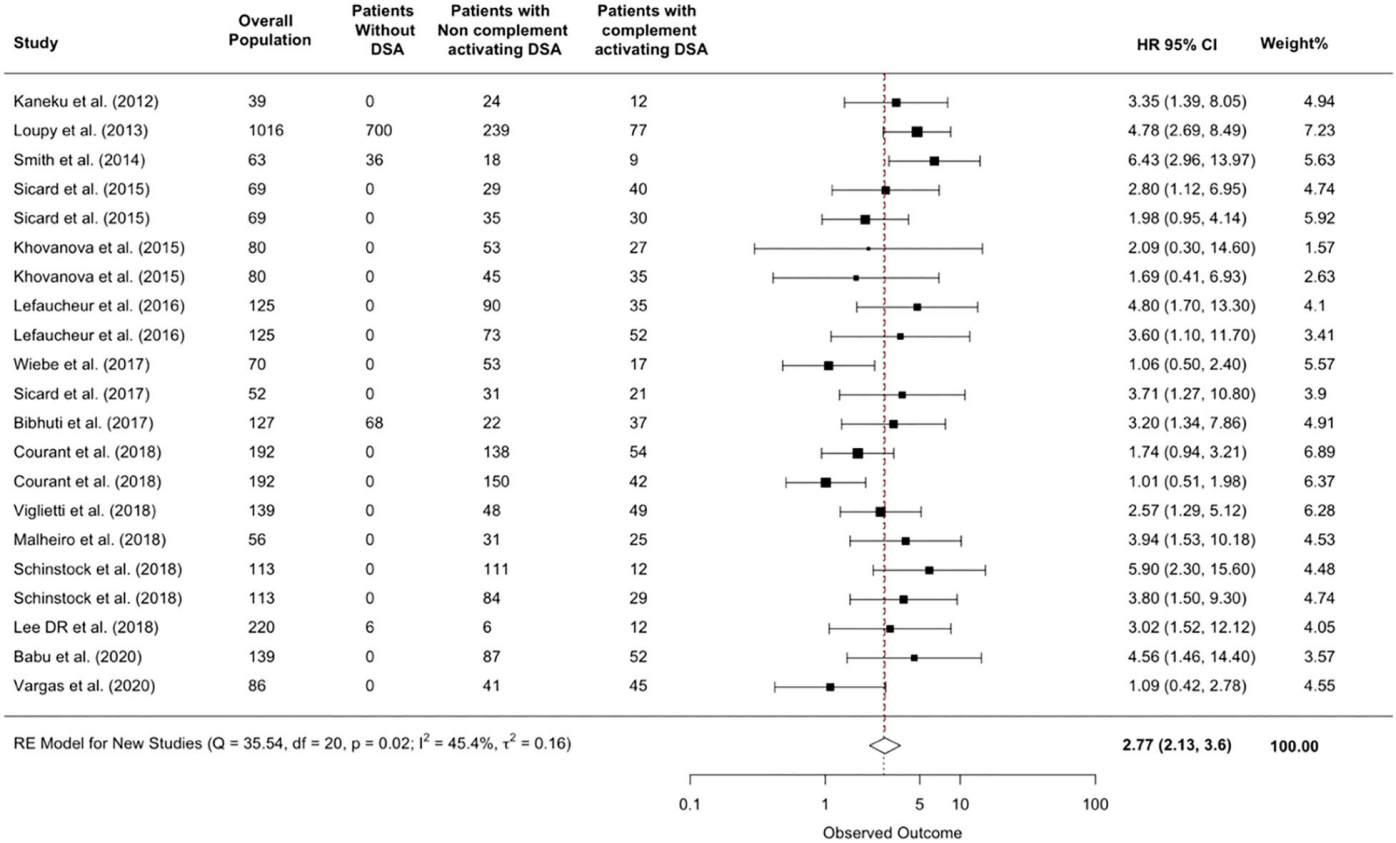
Added prognostic value of complement-activating anti-HLA DSA status over anti-HLA DSA MFI level on allograft loss. The figure shows the forest plot of the association between complement-activating anti-HLA DSAs and the risk of allograft loss for each c study that included the anti-HLA DSA MFI level in their evaluation of the prognostic value of complement-activating anti-HLA DSA. Number of patients are listed in the 4 cohort columns. The black square-shaped boxes represent the HR for each individual study. The size of these boxes represents the weight of the study, and lines represent the 95% CI for individual studies. The diamond at the bottom represents the pooled HR. CI, confidence interval; DSA, donor-specific antibody; HLA, human leukocyte antigen; HR, hazard ratio.

26 (50%) studies reported positive correlation between complement-activating anti-HLA DSA and pan-IgG anti-HLA DSA level defined by the MFI. 15 (37.5%) studies performed multivariable analyses adjusting complement-activating anti-HLA DSA status on pan-IgG anti-HLA DSA defined by the MFI levels as opposed to a linear univariable correlation analysis. The multivariable analysis demonstrated that complement-activating anti-HLA DSA’s presence was significantly and independently associated with an increased risk of allograft loss (HR 2.77; 95% CI 2.13-3.6, p=0.017; I^2 ^= 45.4%) (**Figure 6**).

Seven (13.5%) studies pre-treated the sera of the studied population, or a sample of the studied population, with EDTA to uncover interfering substances and only 3 studies (5.8%) performed a multivariable analysis models adjusting complement-activating anti-HLA DSA status on EDTA treated pan-IgG anti-HLA DSA assays.

### 3.9 Added prognostic value of complement-activating anti-HLA DSA status over anti-HLA DSA class type

Among the 29 (55.8%) studies that used multivariate analysis to evaluate the risk of allograft loss, only three (5.8%) studies included DSA class as a predictive variable. Among these three studies, two showed that HLA class II DR was significantly associated with graft loss. Complement activating anti-HLA DSA remained independently associated with an increased risk for graft loss HR=3.76 (CI=2.33-6.06; p=0.626; I2 = 0%) however, the results were statistically insignificant due to the low number of studies that included DSA type in the multivariable models.

## 4 Discussion

### 4.1 Study overview

In this systematic review, meta-analysis, and critical appraisal including 11,035 solid organ recipients, we confirmed the increased risk of allograft failure and rejection associated with complement-binding anti-HLA DSAs. To the best of our knowledge, this is the first comprehensive systematic review and meta-analysis on the topic and the first in-depth critical appraisal assessing for the risk of bias, adjusting for it and providing several subgroup analyses to study the association of complement-binding anti-HLA DSAs with allograft outcomes. We also addressed the utility of complement-activating anti-HLA DSAs assessment over anti-HLA DSA MFI levels.

### 4.2 Subgroup analyses findings

This meta-analysis showed consistent results in multiple subgroup analyses. Complement-activating anti-HLA DSA were associated with an increased risk for allograft loss in higher quality studies, in different types of complement-activating anti-HLA DSAs (C1q, C3d, C4d and IgG3), at different times of evaluation for complement-activating anti-HLA DSA status (before and after transplantation) and at different MFI thresholds for complement-activating anti-HLA DSA positivity.

### 4.3 Cumulative meta-analysis findings

The cumulative meta-analysis further illustrated the significant overall effect of complement activating anti-HLA DSAs on allograft loss. Combining this finding with our findings from the subgroup analyses, we can perceive saturation of knowledge in particular in kidney transplant recipients and C1q evaluations. This is due to the fact that the majority of patients assessed were kidney recipients (78%) who were tested for C1q (54%) and therefore further research in this particular area could be redundant. However, there remains some areas that could benefit from further exploration, for instance, we did not identify any studies on the effect of complement-binding anti-HLA DSAs in pancreas and intestine transplants. In addition, more studies in liver, lung and heart recipients could be beneficial to confirm the initial findings by increasing the sample size and by comparing the risk of allograft loss across different organ transplants.

### 4.4 Added prognostic value of complement-activating anti-HLA DSA status over anti-HLA DSA MFI level

Several studies in this meta-analysis and in the literature (53, 71, 72) indicated a strong correlation between complement-activating antibody status and anti-HLA DSA MFI level. Interestingly, studies included in this meta-analysis that performed multivariable analyses for the assessment of the independent prognostic value of complement-activating anti-HLA DSA adjusted on pan-IgG anti-HLA DSA defined by the MFI levels, showed that the association between C1q, C3d, C4d-binding tests or IgG3 test and allograft lost was independent of anti-HLA DSA MFI levels.

Although the absence of DSA complement binding antibodies should not be considered as a lack of the harmful effects of DSA *in vivo*, our meta-analysis supports a clinical utility of performing complement-binding assays. Indeed, the clinical impact remains significantly associated with graft loss independent of anti-HLA DSA MFI levels.

In addition to the uncertain association between the MFI levels and the clinical significance of an antibody, SAB pan-IgG assay remains a semi-quantitative test and technical limitations have been raised such as significant variations in repeated testing, between different laboratories (73), and due to various interfering substances (74). In addition, even though some studies addressed interfering substances by pretreatment of sera with EDTA (12, 13, 50, 53, 59), several limitations were noted; the EDTA concentrations were inconsistent across the studies, two studies only pretreated a small sample of the studied populations (4-8 patients), and the prognostic advantage of EDTA treated sera over complement assays was not demonstrated.

Therefore, our study shows that the use of complement binding anti-HLA DSA in clinical practice, in complement to MFI levels, which remains gold standard, could enhance risk stratification.

### 4.5 Added prognostic value of complement-activating anti-HLA DSA status over anti-HLA DSA class

We could not show independent association of complement-activating anti-HLA DSA status over HLA-DSA class due insufficient data published so far (only 3 studies). Further studies should therefore investigate the independent impact of class I or class II anti-HLA DSA regardless of their ability to activate complement, but also investigate the clinical impact of class I versus class II complement-activating anti-HLA DSA.

### 4.6 Implications

This study addresses several gaps highlighted by the STAR working group including the strong evidence regarding the prognostic role of complement-activating anti-HLA DSA in allograft rejection and loss, in complement to HLA-DSA titre and MFI assessment. This strongly supports a potential role for this test in clinical practice. and encourages interventional research regarding the role of certain drugs that target complement-dependent cytotoxicity as a prophylaxis and/or treatment of antibody-mediated rejection and the value of a complement-activating anti-HLA DSA based strategy to monitor organ transplant patients to demonstrate clinical benefit and improvement of allograft survival.

### 4.7 Limitations

This study has the following limitations. First, we only included studies that provided a clear effect size for allograft loss or rejection (hazard or odds ratio). Second, No data was available from South America, Africa and Australia to reinforce the generalizability of the results. Third, all of the included studies were observational and retrospective. Finally, the review only included studies written in English.

## 5 Conclusion

The results of this systematic review, meta-analysis and critical appraisal support the significant and independent detrimental effects of complement-activating anti-HLA DSAs on allograft outcomes. This study highlights areas that need further exploration in complement-activating anti-HLA DSAs research, and encourages the clinical evaluation of complement-activating anti-HLA DSA testing to improve risk stratification and tailoring treatment regimens.

## Data availability statement

The raw data supporting the conclusions of this article will be made available upon reasonable request.

## Author contributions

S-AA: Conceptualization, Data curation, Formal analysis, Investigation, Methodology, Software, Writing- original draft, Writing- review & editing. MR: Formal analysis, Methodology, Supervision, Validation, Writing- review & editing. KL: Methodology, Supervision, Validation, Writing- review & editing. AB: Data curation, Formal analysis, Investigation, Methodology, Validation, Writing- original draft, Writing- review & editing. J-LT: Validation, Writing- review & editing. OA: Methodology, Validation, Writing- review & editing. AL: Conceptualization, Methodology, Project administration, Supervision, Validation, Writing- review & editing. CL: Conceptualization, Funding acquisition, Methodology, Project administration, Resources, Supervision, Writing- review & editing.
