# Supplementary material for: Complement-activating donor-specific anti-HLA antibodies in solid organ transplantation: systematic review, meta-analysis, and critical appraisal
Source: Front Immunol. 2023 Oct 2;14:1265796. doi: 10.3389/fimmu.2023.1265796 (PMC10577173; doi:10.3389/fimmu.2023.1265796)
Supplement: Supplementary file 1 [file DataSheet_1.docx]

Supplementary Material

1. **Supplementary Methods**

Protocol of the study

Search strategy PubMed

Quality assessment of non-randomized trial

Heterogeneity and Publication bias

**Supplementary Tables**

Appendix Table 1: Methodological quality assessment of non-randomized trials

Appendix Table 2: Association between circulating complement-activating anti-HLA DSA and graft loss per organ type.

**Supplementary Figures**

Appendix Figure 1: Association between circulating complement-activating anti-HLA DSAs and the risk of allograft loss in the newly identified studies.

Appendix Figure 2: Association between circulating complement-activating anti-HLA DSAs and the risk of allograft rejection in the newly identified studies.

Appendix Figure 3: Adjustment for small-size effects by PET method

Appendix Figure 4: Cumulative meta-analysis forest plot

**References**

**Supplementary methods:**

1. *Protocol of the study*

Review question

Is there a change in associated risk between complement-activating anti-human leucocyte antigen donor specific antibodies and graft survival or episodes of rejection in solid organ transplant patients since the publication of the original review by *Bouquegneau et al.(1)?*

Specific aims

Aim 1)

To update the results of the original systematic review and meta-analysis by Bouquegneau *et al.(1)* by including the newly identified studies assessing the effect of circulating complement-activating anti-HLA DSAs on allograft outcomes.

Aim 2)

To improve the results of the updated systematic review and meta-analysis by further evaluating and adjusting for risk of bias.

**Hypothesis:**

Circulating complement-activating anti-HLA DSAs are associated with a lower graft survival and an increased risk of antibody mediated rejection compared to patients with circulating anti-HLA DSAs without the capability of activating the complement.

**Relevance of the proposed research:**

Since the publication of the original review, more studies continue to be conducted evaluating the role of complement activating anti-HLA DSAs and the graft survival or rejection appearance. This update will provide a more comprehensive analysis of the available literature. Depending on findings, this will aid the assessment of complement activating anti-HLA DSAs in the patient management and also guide the treatment.

Eligibility criteria

INCLUSION

**Study designs:**Case-crossover study, case control study, cohort study, randomized control study and in English language.

**Participants:**

Solid organ transplant population: kidney, heart, liver, lung and intestines (small bowel) transplant recipients.

Either adult or paediatric patients.

**Measurement:**

Donor specific anti-human leukocyte antigen antibodies by luminex solid-phase assay technic.

Complement activating anti-HLA DSA (C1q, C3d, C4d) and/or IgG subclass.

**Outcomes of interest:**

Graft survival/loss and/or rejection.

Graft survival/loss is defined as a patient’s definitive return to dialysis or pre-emptive kidney re-transplantation.

Graft rejection is defined as either antibody-mediated or mixed-rejection diagnosed using Banff international classification for kidney and liver transplants (2,3) or the International Society for Heart and Lung Transplantation (ISHLT) classification for heart and lung transplants (4).

EXCLUSION

Animal, ex vivo and methodological studies.

Search Strategy

**Years:** January 31, 2018 and May 5, 2023

**Databases:** PubMed

**Search terms:** “kidney transplantation” “liver transplantation” “lung transplantation” “heart transplantation” “intestine transplantation” “complement binding DSA” “complement activating DSA” “C1q” “C3d” “C4d” “IgG subclass”.

In addition, we will review similar articles of the 3 largest and 3 newest studies of the original review. We also request potentially eligible studies from content experts.

Selection

Two reviewers (S. Al Awadhi and A. Bouquegneau) will consider the potential eligibility of each of the abstracts and titles that result from executing the search strategy. Reviewers will request the full text versions of all potentially eligible studies. Disagreements will be solved by consensus.

Two reviewers (S. Al Awadhi and A. Bouquegneau) working independently and blindly will consider the full text reports (all available versions of each study) for eligibility. The reviewers will calibrate their judgments using a smaller set of reports. Subsequently, disagreements will be harmonized by consensus.

Extraction

Data extraction will include details from study population, author name, year of publication, study size, mean or median follow-up time, type of complement-activating anti-HLA DSAs, comparison used (patients with complement-activating anti-HLA DSAs compared either to patients without complement-activating DSAs, patients without DSAs detected, or a matching group of patients without DSAs and without complement-activating DSAs), effect sizes (HR and/or OR), 95% confidence intervals (CIs), potential confounding factors, and unadjusted and adjusted estimated risks of graft loss or graft rejection. Adjusted HRs and ORs will be used when available; otherwise, univariate effect sizes will be used.

Quality

To assess the methodological quality of studies, we will determine:

- The use of single-antigen flow bead assays to detect donor-specific anti-HLA antibodies and complement-activating DSA
- The level of mean fluorescence intensity (MFI) considered as positive
- Time of follow up
- Validated criteria of rejection according to Banff (3,5) or ISHLT classification (4,6)
- Allograft loss have been or not adjusted/censored for death

Finally, we will ensure that items of the Newcastle-Ottawa quality assessment Scale (NOS) will be verified to quantify studies quality for observational studies (7). Jadad scale will be used to assess a quality of randomized controlled trials (8).

Pooling

When possible, we will generate meta-analytic estimates of each complement activating DSA types on the occurrence of graft loss and rejection (relative risk, odd-ratio and percentage change). As a rule we will use random-effects meta-analyses and measure inconsistency for each outcome by estimating the I^2^ test and its confidence interval (9). We will conduct a cumulative meta-analysis to evaluate the impact each new study has on the overall effect size (10).

We will use several available software packages to conduct the analyses (R software version 4.1.1).

Subgroups

To explore causes of inconsistency and subgroup-confounders interactions, we will construct the following subgroup analyses defined by:

1. High methodological quality studies vs low methodological quality: articles with NOS scores ≥6 (vs. lower scores) were selected as high-quality studies
2. Type of organ transplanted: kidney allograft vs. all other types of transplanted organs (heart, lung and liver allografts)
3. Timing of antibody detection: pre-existing anti-HLA DSAs, defined as antibodies present before or at the time of transplantation, or *de novo* anti-HLA DSAs, defined as antibodies present only after transplantation, or a combined group of pre-existing and *de novo* DSAs
4. Type of assay used for characterizing the complement-activating capacity of antibodies: assays were characterised as anti-HLA DSA IgG subclass, C1q-binding anti-HLA DSAs, C4d-binding anti-HLA DSAs, or C3d-binding anti-HLA DSAs

We will measure the difference in effect sizes between subgroups (univariate analyses). When possible, we will construct meta-regression analyses with subgroups as the independent variables and outcomes as the dependent variable.

Reporting heterogeneity and bias

Statistical heterogeneity across the studies will be tested with the I^2^ index (11). The I^2^ index describes the percentage of total variation across studies due to heterogeneity rather than chance. A value of 0% indicates no observed heterogeneity, values exceeding 50% may elicit considerable caution and warrant further analysis through subgroup analyses (12).

Small study effects and publication bias will be visually assessed using funnel plots and statistically assessed by the Egger’s bias coefficient, which weighted the regression of the intervention effect on its standard error (SE), with weights inversely proportional to the variance of the intervention effect (13). Risk of bias adjustment methods will be used when appropriate(14).

1. *Search strategy*

Database: Pubmed

Strategy: Complementary search strategy: Narrow Boolean search and ranking strategies(15).

| Searches | Results |
| --- | --- |
| **Narrow Boolean search strategy** |  |
| (((((((((kidney transplantation[MeSH Terms]) OR (liver transplantation[MeSH Terms])) OR (heart transplantation[MeSH Terms])) OR (lung transplantation[MeSH Terms])) OR (intestine transplantation[MeSH Terms])) OR (pancreas transplantation[MeSH Terms]) ) AND (c4 complement component[MeSH Terms])) OR (complement, c1q[MeSH Terms])) OR (c3d complement[MeSH Terms])) OR (igg3[MeSH Terms]) | 161,465 |
| exp date <2018 | 19,919 |
| exp non-human | 16,838 |
| exp non-English | 16,346 |
| exp “review”, “systematic review”, “meta-Analysis”, “books and documents” | 1,186 |
| **Ranking search: Similar articles of the 3 largest and 3 newest studies of the original review** | 1044 |
| exp date <2018 | 403 |
| exp non-human | 307 |
| exp non-English | 303 |

1. *Quality assessment of non-randomized trial*

The Newcastle-Ottawa Scale (NOS) was used to assess the quality of the non-randomized studies included in the systematic review and meta-analysis (7). Using this quality score, each study is judged on eight items categorized into the following 3 major components: selection of the study groups and ascertainment (0 to 4 points), quality of the adjustments for confounding variables (0 to 2 points), and outcomes (0 to 3 points). Stars awarded for each quality item serve as a quick visual assessment. Stars are awarded such that the highest quality studies are awarded up to nine stars. A higher score represents better methodological quality (16).

The 3 components are described below:

1. Selection of the study groups
   1. Representativeness of the exposed cohort
      1. Truly representative of the average in the community *****
      2. Somewhat representative of the average in the community *****
      3. Selected group of users, e.g., nurses, volunteers
      4. No description of the derivation of the cohort
   2. Selection of the non-exposed cohort
      1. Drawn from the same community as the exposed cohort *****
      2. Drawn from a different source
      3. No description of the derivation of the non-exposed cohort
   3. Ascertainment of exposure
      1. Secure record (e.g., surgical records) *****
      2. Structured interview *****
      3. Written self-report
      4. No description
   4. Demonstration that the outcome of interest was not present at the start of the study (for example, surveillance biopsy in cases of rejection evaluation)
      1. Yes*****
      2. No
2. Comparability
   1. Comparability of the cohorts on the basis of the design or analysis
      1. Study controls for certain statistical parameters (over-fitting of statistical models, not enough events, variables included in multivariate models) *****
      2. Study controls for any additional factors ***** (description for not using multivariate models)
3. Outcomes
   1. Assessment of outcomes
      1. Independent blind assessment *****
      2. Record linkage *****
      3. Self-report
      4. No description
   2. Sufficient follow-up for outcomes
      1. Yes (5-years) *****
      2. No
   3. Adequacy of the follow-ups for cohorts
      1. Complete follow-up - all subjects accounted for *****
      2. Subjects lost to follow-up who are unlikely to introduce bias – a small number lost *****
      3. Follow up rate < 80% and no description of those lost
      4. No statement
4. *Heterogeneity and Publication bias*

Statistical heterogeneity across the studies was tested with the I^2^ index (11). The I^2^ index describes the percentage of total variation across studies due to heterogeneity rather than chance. A value of 0% indicates no observed heterogeneity, values exceeding 50% may elicit considerable caution and warrant further analysis through subgroup analyses (12).

Publication bias was assessed using funnel plots and the Egger’s bias coefficient, which weighted the regression of the intervention effect on its standard error (SE), with weights inversely proportional to the variance of the intervention effect (13). Methods to adjust for risk of bias will be used if appropriate.

**Appendix Table 1: Methodological quality assessment of non-randomized trials** New Castle-Ottawa Scale evaluation of the studies included in the meta-analysis (n=52), stratified by organ type. Abbreviations: C1q, complement component 1q; CI, confidence interval; DSA, donor-specific antibody; IgG3, immunoglobulin G3.

| **Authors (years) (reference)** | **Type of study** | **Type of C’ anti-HLA DSA (Organ)** | **NOS Classification** | | |
| --- | --- | --- | --- | --- | --- |
|  |  |  | **Selection** | **Comparability** | **Outcome** |
| Sutherland et al. (2011) (17) | Cohort | C1q (Kidney) | *** | ** | ** |
| Freitas et al. (2013) (18) | Cohort | IgG3/C1q (Kidney) | **** | * | ** |
| Loupy et al. (2013) (19) | Cohort | C1q (Kidney) | **** | ** | ** |
| Crespo et al. (2013) (20) | Cohort | C1q (Kidney) | **** |  | * |
| Thammanichanond et al. (2016) (21) | Cohort | C1q (Kidney) | *** |  | * |
| Fichtner et al. (2016) (22) | Cohort | C1q (Kidney) | **** | * | *** |
| Calp-Inal et al. (2016) (23) | Cohort | C1q (Kidney) | *** | * | * |
| Guidicelli et al. (2016) (24) | Cohort | C1q (Kidney) | **** | * | *** |
| Bamoulid et al. (2016) (25) | Cohort | C1q (Kidney) | *** |  | *** |
| Kauke et al. (2016) (26) | Cohort | C1q (Kidney) | **** | * | ** |
| Yamamoto et al. (2016) (27) | Cohort | C1q (Kidney) | *** | * | ** |
| Moktefi A. et al. (2017) (28) | Cohort | C1q (Kidney) | *** | * | * |
| Wiebe et al. (2017) (29) | Cohort | C1q (Kidney) | *** | * | ** |
| Malheiro et al. (2017) (30) | Cohort | C1q (Kidney) | **** | * | * |
| Sicard et al. (2015) (31) | Cohort | C3d/C1q (Kidney) | **** | * | * |
| Comoli et al. (2016) (32) | Cohort | C3d/C1q (Kidney) | *** |  | * |
| Sicard et al. (2017) (33) | Cohort | C3d (Kidney) | *** | ** | ** |
| Wahrmann et al. (2009) (34) | Cohort | C4d (Kidney) | *** | * | ** |
| Hönger et al. (2010) (35) | Cohort | C4d (Kidney) | *** | * | * |
| Bartel et al. (2013) (36) | Cohort | C4d (Kidney) | *** | * | ** |
| Lawrence et al. (2013) (37) | Cohort | C4d (Kidney) | *** | * | * |
| Hönger et al. (2011) (38) | Cohort | IgG3 (Kidney) | *** | * | * |
| Arnold et al. (2014) (39) | Cohort | IgG3 (Kidney) | **** |  | * |
| Everly et al. (2014) (40) | Cohort | IgG3 (Kidney) | *** | * | ** |
| Khovanova et al. (2015) (41) | Cohort | IgG3 (Kidney) | *** | * | ** |
| Lefaucheur et al. (2016) (42) | Cohort | IgG3/C1q (Kidney) | **** | ** | * |
| Viglietti et al. (2017) (43) | Cohort | IgG3/C1q (Kidney) | **** | ** | *** |
| Smith J. et al. (2011) (44) | Cohort | C4d (Heart) | **** | * | * |
| Bibhuti B. et al. (2017) (45) | Cohort | C1q (Heart) | **** | ** | * |
| Kaneku H. et al. (2012) (46) | Case control | IgG3 (Liver) | *** | ** | * |
| O’Leary J. et al. (2015) (47) | Cohort | IgG3/C1q (Liver) | **** | * | * |
| Wozniak L. et al. (2015) (48) | Cohort | C1q (Liver) | * |  | ** |
| Couchonnal et al. (2017) (49) | Cohort | C3d (Liver) | **** | ** | * |
| Smith J. et al. (2014) (50) | Cohort | C4d (Lung) | **** | * | * |
| Visentin J. et al. (2016) (51) | Cohort | C1q (Lung) | **** | * | * |
| Lan et al. (2018) (52) | Cohort | C3d (Kidney) | **** | * | ** |
| Courant et al. (2018) (53) | Cohort | C1q/C3d (Kidney) | *** | ** | * |
| Brugière et al. (2018) (54) | Cohort | C1q (Lung) | **** | ** | ** |
| Kamburova et al. (2018) (55) | Cohort | C1q (Kidney) | **** | ** | ** |
| Viglietti et al. (2018) (56) | Cohort | C1q (Kidney) | *** | ** | *** |
| Lee H et al. (2018) (57) | Cohort | C1q/C3d (Kidney) | *** | ** | ** |
| Malheiro et al. (2018) (58) | Cohort | C1q (Kidney) | *** | * | *** |
| Schinstock et al. (2018) (59) | Cohort | C1q/IgG3 (Kidney) | *** | * | * |
| Lee DR et al. (2018) (60) | Cohort | C3d (Kidney) | **** | ** | ** |
| Babu et al. (2020) (61) | Cohort | C3d (Kidney) | ** | * | * |
| Vargas et al. (2020) (62) | Cohort | C1q (Kidney) | ** | * | ** |
| Zhang et al. (2018) (63) | Cohort | C3d (Heart) | **** | * | ** |
| Cioni et al. (2019) (64) | Cohort | C1q/C3d (Kidney) | **** |  | * |
| Hayde et al. (2020) (65) | Cohort | C1q (Kidney) | **** |  | * |
| Pernin et al. (2020) (66) | Cohort | IgG3 (Kidney) | **** | * | * |

**Appendix Table 2: Association between circulating complement-activating anti-HLA DSA with graft loss per organ type.**

| **Organ** | **Number of studies** | **Number of patients** | **HR for Graft loss** |
| --- | --- | --- | --- |
| Kidney | 44 | 8,746 (79.3%) | 2.77 (95% CI 2.25-3.41, p<0.001; I^2^=49.2%). |
| Lung | 4 | 284 (2.6%) | 3.26 (95% CI 1.44-7.34, p=0.073; I^2^=60.7%) |
| Liver | 3 | 1,459 (13.2%) | 2.21 (95% CI 1.65-2.96, p=0.523; I^2^=00.0%) |
| Heart | 1 | 546 (4.9%) | 3.20 (95% CI 1.34-7.80, p=0.009; I^2^=NA) |

Abbreviations: CI, confidence interval; HR, hazard ratio, NA, not applicable.

**Supplementary Figures**

**Appendix Figure 1** Association between circulating complement-activating anti-HLA DSAs and the risk of allograft loss in the newly identified studies . The figure shows forest plot for the newly identified studies since the publication of the previous review in 2018 and the association between complement-activating anti-HLA DSAs and the risk of allograft loss for each complement binding study and overall (n = 10). Studies are listed by date of publication. Number of patients are listed in the 4 cohort columns. The black square-shaped boxes represent the HR for each individual study. The size of these boxes represents the weight of the study, and lines represent the 95% CI for individual studies. The diamond at the bottom represents the pooled HR.

**
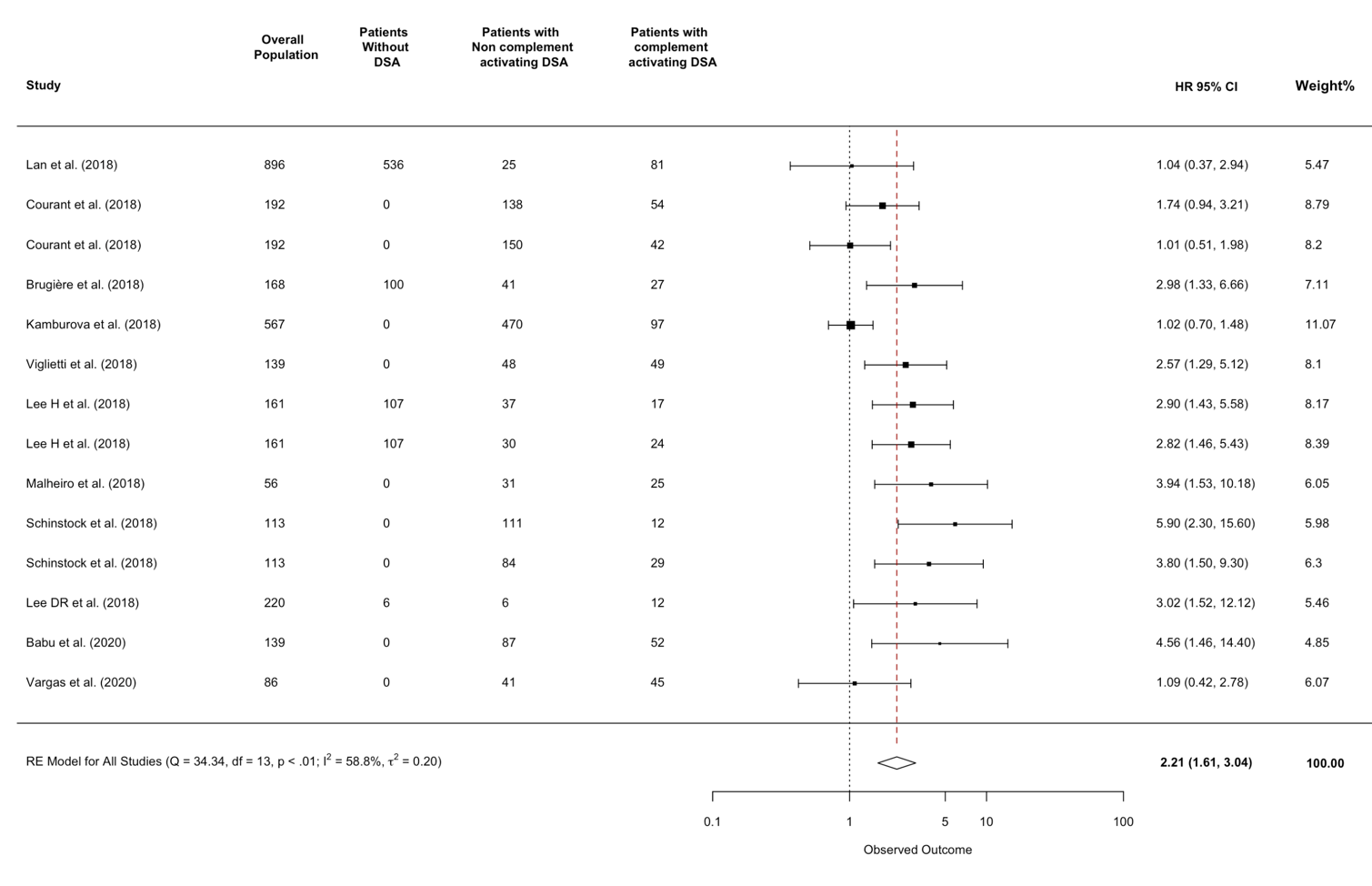
**

**Appendix Figure 2** Association between circulating complement-activating anti-HLA DSAs and the risk of allograft rejection in the newly identified studies. The figure shows forest plot for the newly identified studies since the publication of the previous review in 2018 and the association between complement-activating anti-HLA DSAs and the risk of allograft loss for each complement binding study and overall (n = 5). Studies are listed by date of publication. Number of patients are listed in the 4 cohort columns. The black square-shaped boxes represent the HR for each individual study. The size of these boxes represents the weight of the study, and lines represent the 95% CI for individual studies. The diamond at the bottom represents the pooled HR.

**
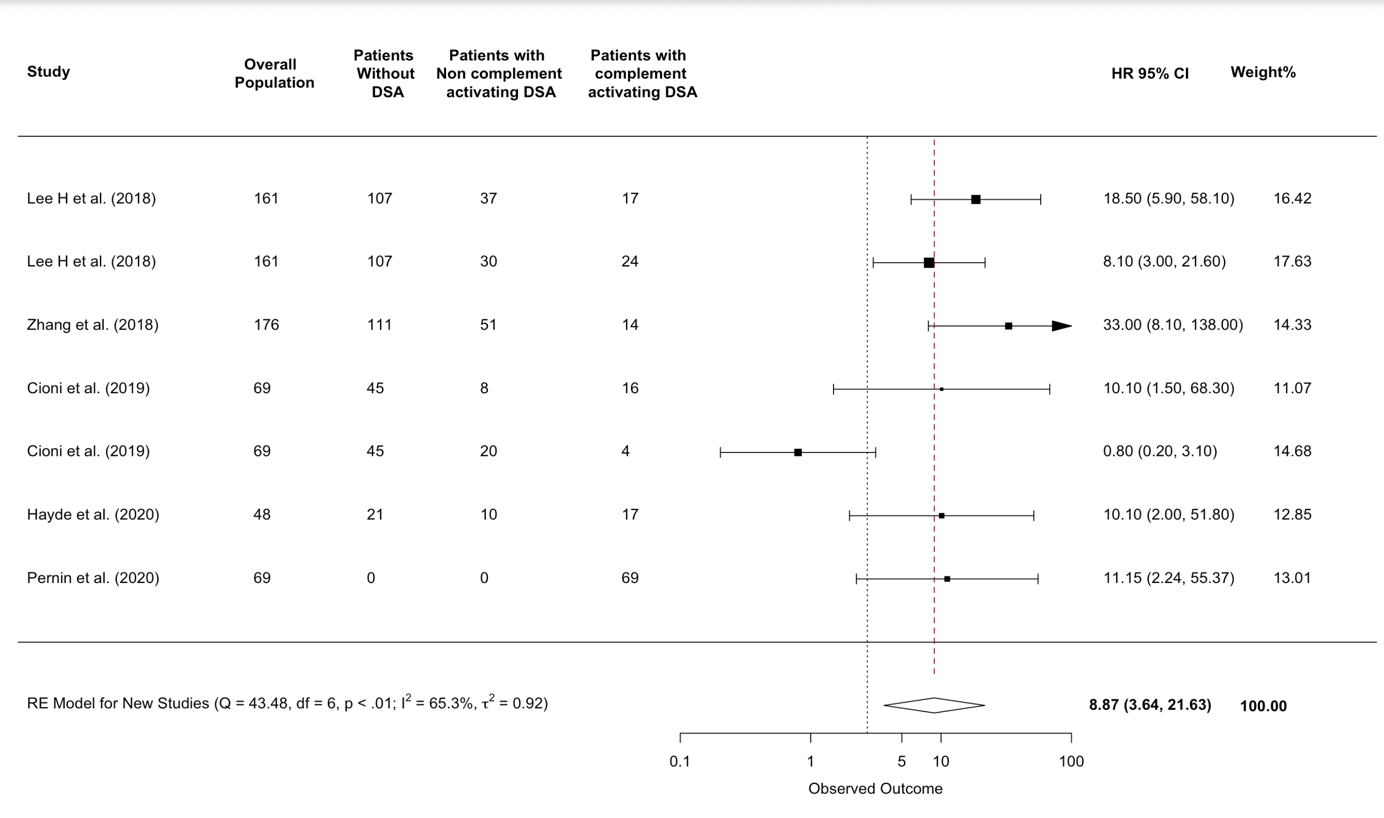
**

**Appendix Figure 3** Adjustment for small-size effects by PET method.

Each black dot represents a study; the x-axis represents the study effect size (HR) and the y-axis represents the standard error of the HR. The red line represents the pooled HR for allograft loss and the blue line represents PET regression line. The blue circle represents the pooled HR in a hypothetical infinite sample size.

Abbreviations: PET, precision-effect test; HR, hazard ratio.


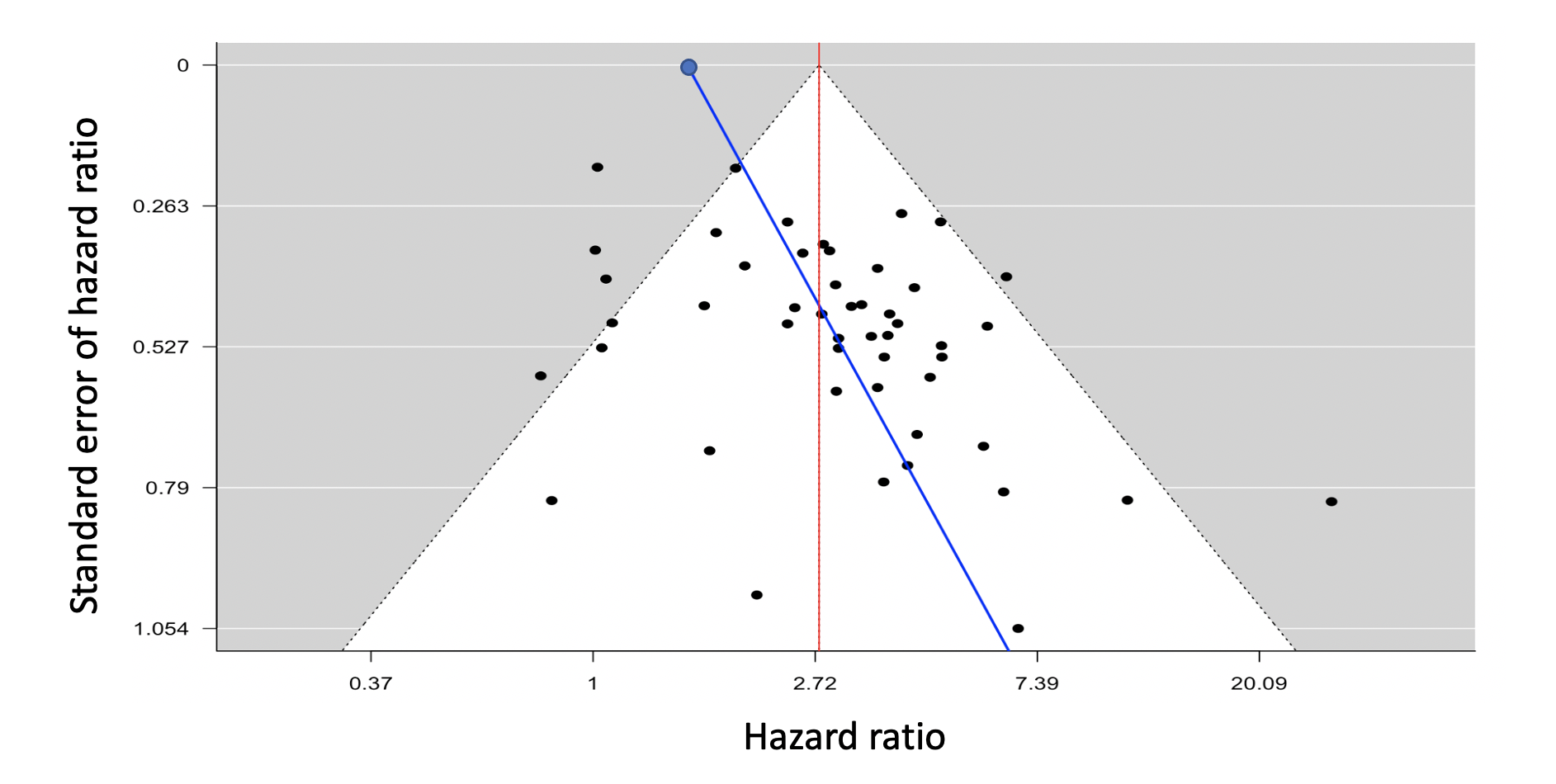


**Appendix Figure 4 Cumulative meta-analysis of the effect of complement-binding anti-HLA DSAs on allograft loss.**  This figure shows the forest plot of the cumulative association between complement activating anti-HLA DSAs and the risk allograft loss. The studies are arranged by date of publication. The black square-shaped boxes represent the HR for each individual study. The quare-shaped box at the end represents the overall HR. HR, hazard ratio


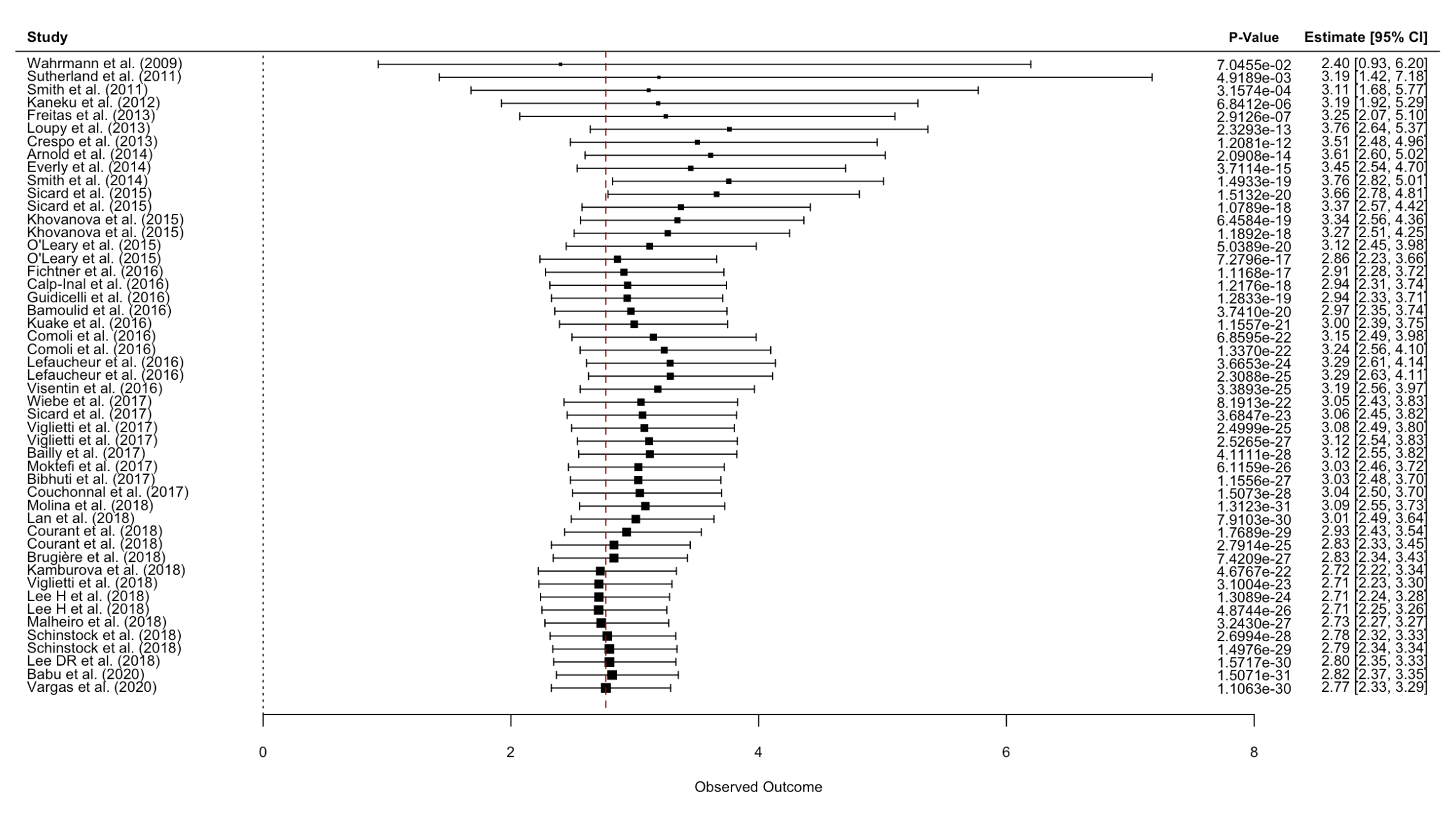


**4) Supplementary references**

1. Bouquegneau A, Loheac C, Aubert O, Bouatou Y, Viglietti D, Empana J, et al. Complement-activating donor-specific anti-HLA antibodies and solid organ transplant survival: A systematic review and meta-analysis. PLoS Med [Internet]. 2018 May 25 [cited 2021 May 25];15(5). Available from: https://www.ncbi.nlm.nih.gov/pmc/articles/PMC5969739/

2. Loupy A, Haas M, Solez K, Racusen L, Glotz D, Seron D, et al. The Banff 2015 Kidney Meeting Report: Current Challenges in Rejection Classification and Prospects for Adopting Molecular Pathology. Am J Transplant Off J Am Soc Transplant Am Soc Transpl Surg. 2017 Jan;17(1):28–41.

3. Demetris A, Adams D, Bellamy C, Blakolmer K, Clouston A, Dhillon AP, et al. Update of the International Banff Schema for Liver Allograft Rejection: working recommendations for the histopathologic staging and reporting of chronic rejection. An International Panel. Hepatology. 2000 Mar;31(3):792–9.

4. Berry GJ, Angelini A, Burke MM, Bruneval P, Fishbein MC, Hammond E, et al. The ISHLT working formulation for pathologic diagnosis of antibody-mediated rejection in heart transplantation: evolution and current status (2005-2011). J Heart Lung Transplant. 2011 Jun;30(6):601–11.

5. Loupy A, Haas M, Solez K, Racusen L, Glotz D, Seron D, et al. The Banff 2015 Kidney Meeting Report: Current Challenges in Rejection Classification and Prospects for Adopting Molecular Pathology. Am J Transplant. 2017 Jan;17(1):28–41.

6. Meyer KC, Raghu G, Verleden GM, Corris PA, Aurora P, Wilson KC, et al. An international ISHLT/ATS/ERS clinical practice guideline: Diagnosis and management of bronchiolitis obliterans syndrome. Eur Respir J. 2014;44(6):1479–503.

7. Wells GA, Shea B, O’Connell D, Peterson J, Welch V, Losos M, et al. The Newcastle-Ottawa Scale (NOS) for assessing the quality of non- randomised studies in meta-analyses. [Internet]. Available from: http://www.ohri.ca/programs/ clinical_epidemiology/oxford.htm. Accessed December, 2016.

8. Jadad AR, Moore RA, Carroll D, Jenkinson C, Reynolds DJ, Gavaghan DJ, et al. Assessing the quality of reports of randomized clinical trials: is blinding necessary? Control Clin Trials. 1996 Feb;17(1):1–12.

9. Nikolakopoulou A, Mavridis D, Salanti G. Demystifying fixed and random effects meta-analysis. Evid Based Ment Health. 2014 May;17(2):53–7.

10. Lau J, Antman EM, Jimenez-Silva J, Kupelnick B, Mosteller F, Chalmers TC. Cumulative Meta-Analysis of Therapeutic Trials for Myocardial Infarction. N Engl J Med. 1992 Jul 23;327(4):248–54.

11. Higgins JPT, Thompson SG, Deeks JJ, Altman DG. Measuring inconsistency in meta-analyses. BMJ. 2003 Sep 6;327(7414):557–60.

12. Higgins JPT, Thompson SG. Quantifying heterogeneity in a meta-analysis. Stat Med. 2002 Jun 15;21(11):1539–58.

13. Egger M, Davey Smith G, Schneider M, Minder C. Bias in meta-analysis detected by a simple, graphical test. BMJ. 1997 Sep 13;315(7109):629–34.

14. Aert RCM van, Wicherts JM, Assen MALM van. Publication bias examined in meta-analyses from psychology and medicine: A meta-meta-analysis. PLOS ONE. 2019 Apr 12;14(4):e0215052.

15. Garner P, Hopewell S, Chandler J, MacLehose H, Akl EA, Beyene J, et al. When and how to update systematic reviews: consensus and checklist. BMJ. 2016 Jul 20;354:i3507.

16. Ayoub C, Erthal F, Abdelsalam MA, Murad MH, Wang Z, Erwin PJ, et al. Prognostic value of segment involvement score compared to other measures of coronary atherosclerosis by computed tomography: A systematic review and meta-analysis. J Cardiovasc Comput Tomogr. 2017 Aug;11(4):258–67.

17. Sutherland SM, Chen G, Sequeira FA, Lou CD, Alexander SR, Tyan DB. Complement-fixing donor-specific antibodies identified by a novel C1q assay are associated with allograft loss. Pediatr Transplant. 2012 Feb;16(1):12–7.

18. Freitas MCS, Rebellato LM, Ozawa M, Nguyen A, Sasaki N, Everly M, et al. The role of immunoglobulin-G subclasses and C1q in de novo HLA-DQ donor-specific antibody kidney transplantation outcomes. Transplantation. 2013 May 15;95(9):1113–9.

19. Loupy A, Lefaucheur C, Vernerey D, Prugger C, Duong van Huyen JP, Mooney N, et al. Complement-binding anti-HLA antibodies and kidney-allograft survival. N Engl J Med. 2013 Sep 26;369(13):1215–26.

20. Crespo M, Torio A, Mas V, Redondo D, Pérez-Sáez MJ, Mir M, et al. Clinical relevance of pretransplant anti-HLA donor-specific antibodies: does C1q-fixation matter? Transpl Immunol. 2013 Dec;29(1–4):28–33.

21. Thammanichanond D, Wiwattanathum P, Mongkolsuk T, Kantachuvesiri S, Worawichawong S, Vallipakorn SA, et al. Role of Pretransplant Complement-fixing Donor-specific Antibodies Identified by C1q Assay in Kidney Transplantation. Transplant Proc. 2016 Apr;48(3):756–60.

22. Fichtner A, Süsal C, Höcker B, Rieger S, Waldherr R, Westhoff JH, et al. Association of C1q-fixing DSA with late graft failure in pediatric renal transplant recipients. Pediatr Nephrol. 2016 Jul;31(7):1157–66.

23. Calp-Inal S, Ajaimy M, Melamed ML, Savchik C, Masiakos P, Colovai A, et al. The prevalence and clinical significance of C1q-binding donor-specific anti-HLA antibodies early and late after kidney transplantation. Kidney Int. 2016 Jan;89(1):209–16.

24. Guidicelli G, Guerville F, Lepreux S, Wiebe C, Thaunat O, Dubois V, et al. Non-Complement-Binding De Novo Donor-Specific Anti-HLA Antibodies and Kidney Allograft Survival. J Am Soc Nephrol. 2016 Feb;27(2):615–25.

25. Bamoulid J, Roodenburg A, Staeck O, Wu K, Rudolph B, Brakemeier S, et al. Clinical Outcome of Patients with De Novo C1q-Binding Donor-Specific HLA Antibodies after Renal Transplantation: Transplantation. 2017 Sep;101(9):2165–74.

26. Kauke T, Oberhauser C, Lin V, Coenen M, Fischereder M, Dick A, et al. De novo donorspecific anti-HLA antibodies after kidney transplantation are associated with impaired graft outcome independently of their C1q-binding ability. Transpl Int. 2017 Apr;30(4):360–70.

27. Yamamoto T, Watarai Y, Takeda A, Tsujita M, Hiramitsu T, Goto N, et al. De Novo Anti-HLA DSA Characteristics and Subclinical Antibody-Mediated Kidney Allograft Injury. Transplantation. 2016;100(10):2194–202.

28. Moktefi A, Parisot J, Desvaux D, Canoui-Poitrine F, Brocheriou I, Peltier J, et al. C1q binding is not an independent risk factor for kidney allograft loss after an acute antibody-mediated rejection episode: a retrospective cohort study. Transpl Int. 2017 Mar;30(3):277–87.

29. Wiebe C, Gareau AJ, Pochinco D, Gibson IW, Ho J, Birk PE, et al. Evaluation of C1q Status and Titer of *De Novo* Donor-Specific Antibodies as Predictors of Allograft Survival. Am J Transplant. 2017 Mar;17(3):703–11.

30. Malheiro J, Tafulo S, Dias L, Martins LS, Fonseca I, Beirão I, et al. Determining donor-specific antibodies C1q-binding ability improves the prediction of antibody-mediated rejection in HLA-incompatible kidney transplantation. Transpl Int. 2017 Apr;30(4):347–59.

31. Sicard A, Ducreux S, Rabeyrin M, Couzi L, McGregor B, Badet L, et al. Detection of C3d-binding donor-specific anti-HLA antibodies at diagnosis of humoral rejection predicts renal graft loss. J Am Soc Nephrol. 2015 Feb;26(2):457–67.

32. Comoli P, Cioni M, Tagliamacco A, Quartuccio G, Innocente A, Fontana I, et al. Acquisition of C3d-Binding Activity by *De Novo* Donor-Specific HLA Antibodies Correlates With Graft Loss in Nonsensitized Pediatric Kidney Recipients. Am J Transplant. 2016 Jul;16(7):2106–16.

33. Sicard A, Meas-Yedid V, Rabeyrin M, Koenig A, Ducreux S, Dijoud F, et al. Computer-assisted topological analysis of renal allograft inflammation adds to risk evaluation at diagnosis of humoral rejection. Kidney Int. 2017 Jul;92(1):214–26.

34. Wahrmann M, Bartel G, Exner M, Regele H, Körmöczi GF, Fischer GF, et al. Clinical relevance of preformed C4d-fixing and non-C4d-fixing HLA single antigen reactivity in renal allograft recipients. Transpl Int. 2009 Oct;22(10):982–9.

35. Hönger G, Wahrmann M, Amico P, Hopfer H, Böhmig GA, Schaub S. C4d-fixing capability of low-level donor-specific HLA antibodies is not predictive for early antibody-mediated rejection. Transplantation. 2010 Jun 27;89(12):1471–5.

36. Bartel G, Wahrmann M, Schwaiger E, Kikic Z, Winzer C, Horl WH, et al. Solid phase detection of C4d-fixing HLA antibodies to predict rejection in high immunological risk kidney transplant recipients. Transpl Int. 2013;26(2):121–30.

37. Lawrence C, Willicombe M, Brookes PA, Santos-Nunez E, Bajaj R, Cook T, et al. Preformed complement-activating low-level donor-specific antibody predicts early antibody-mediated rejection in renal allografts. Transplantation. 2013 Jan 27;95(2):341–6.

38. Hönger G, Hopfer H, Arnold ML, Spriewald BM, Schaub S, Amico P. Pretransplant IgG subclasses of donor-specific human leukocyte antigen antibodies and development of antibody-mediated rejection. Transplantation. 2011 Jul 15;92(1):41–7.

39. Arnold ML, Ntokou IS, Doxiadis IIN, Spriewald BM, Boletis JN, Iniotaki AG. Donor-specific HLA antibodies: evaluating the risk for graft loss in renal transplant recipients with isotype switch from complement fixing IgG1/IgG3 to noncomplement fixing IgG2/IgG4 anti-HLA alloantibodies. Transpl Int. 2014;27(3):253–61.

40. Everly MJ, Rebellato LM, Haisch CE, Briley KP, Bolin P, Kendrick WT, et al. Impact of IgM and IgG3 anti-HLA alloantibodies in primary renal allograft recipients. Transplantation. 2014;97(5):494–501.

41. Khovanova N, Daga S, Shaikhina T, Krishnan N, Jones J, Zehnder D, et al. Subclass analysis of donor HLA-specific IgG in antibody-incompatible renal transplantation reveals a significant association of IgG4 with rejection and graft failure. Transpl Int. 2015 Dec;28(12):1405–15.

42. Lefaucheur C, Viglietti D, Bentlejewski C, Huyen JPD van, Vernerey D, Aubert O, et al. IgG Donor-Specific Anti-Human HLA Antibody Subclasses and Kidney Allograft Antibody-Mediated Injury. J Am Soc Nephrol. 2016 Jan 1;27(1):293–304.

43. Viglietti D, Loupy A, Vernerey D, Bentlejewski C, Gosset C, Aubert O, et al. Value of Donor–Specific Anti–HLA Antibody Monitoring and Characterization for Risk Stratification of Kidney Allograft Loss. J Am Soc Nephrol. 2017 Feb;28(2):702–15.

44. Smith JD, Banner NR, Hamour IM, Ozawa M, Goh A, Robinson D, et al. De novo donor HLA-specific antibodies after heart transplantation are an independent predictor of poor patient survival. Am J Transplant. 2011 Feb;11(2):312–9.

45. Das BB, Lacelle C, Zhang S, Gao A, Fixler D. Complement (C1q) Binding De Novo Donor Specific Antibodies and Cardiac-Allograft Vasculopathy in Pediatric Heart Transplant Recipients. Transplantation. 2017 Sep 6;

46. Kaneku H, O’Leary JG, Taniguchi M, Susskind BM, Terasaki PI, Klintmalm GB. Donor-specific human leukocyte antigen antibodies of the immunoglobulin G3 subclass are associated with chronic rejection and graft loss after liver transplantation. Liver Transpl. 2012 Aug;18(8):984–92.

47. O’Leary JG, Kaneku H, Banuelos N, Jennings LW, Klintmalm GB, Terasaki PI. Impact of IgG3 subclass and C1q-fixing donor-specific HLA alloantibodies on rejection and survival in liver transplantation. Am J Transplant. 2015 Apr;15(4):1003–13.

48. Wozniak LJ, Hickey MJ, Venick RS, Vargas JH, Farmer DG, Busuttil RW, et al. Donor-specific HLA Antibodies Are Associated With Late Allograft Dysfunction After Pediatric Liver Transplantation. Transplantation. 2015;99(7):1416–22.

49. Couchonnal E, Rivet C, Ducreux S, Dumortier J, Bosch A, Boillot O, et al. Deleterious impact of C3d-binding donor-specific anti-HLA antibodies after pediatric liver transplantation. Transpl Immunol. 2017 Aug 3;

50. Smith JD, Ibrahim MW, Newell H, Danskine AJ, Soresi S, Burke MM, et al. Pre-transplant donor HLA-specific antibodies: characteristics causing detrimental effects on survival after lung transplantation. J Heart Lung Transplant. 2014 Oct;33(10):1074–82.

51. Visentin J, Chartier A, Massara L, Linares G, Guidicelli G, Blanchard E, et al. Lung intragraft donor-specific antibodies as a risk factor for graft loss. J Heart Lung Transplant. 2016;35(12):1418–26.

52. Lan JH, Gjertson D, Zheng Y, Clark S, DeKAF Investigators, Reed EF, et al. Clinical utility of complement-dependent C3d assay in kidney recipients presenting with late allograft dysfunction. Am J Transplant Off J Am Soc Transplant Am Soc Transpl Surg. 2018 Dec;18(12):2934–44.

53. Courant M, Visentin J, Linares G, Dubois V, Lepreux S, Guidicelli G, et al. The disappointing contribution of anti-human leukocyte antigen donor-specific antibodies characteristics for predicting allograft loss. Nephrol Dial Transplant. 2018 Oct 1;33(10):1853–63.

54. Brugière O, Roux A, Pavec JL, Sroussi D, Parquin F, Pradère P, et al. Role of C1q-binding anti-HLA antibodies as a predictor of lung allograft outcome. Eur Respir J [Internet]. 2018 Aug 1 [cited 2021 Aug 21];52(2). Available from: https://erj.ersjournals.com/content/52/2/1701898

55. Kamburova EG, Wisse BW, Joosten I, Allebes WA, Meer A van der, Hilbrands LB, et al. Pretransplant C3d-Fixing Donor-Specific Anti-HLA Antibodies Are Not Associated with Increased Risk for Kidney Graft Failure. J Am Soc Nephrol. 2018 Sep 1;29(9):2279–85.

56. Viglietti D, Bouatou Y, Kheav VD, Aubert O, Suberbielle-Boissel C, Glotz D, et al. Complement-binding anti-HLA antibodies are independent predictors of response to treatment in kidney recipients with antibody-mediated rejection. Kidney Int. 2018 Oct 1;94(4):773–87.

57. Lee H, Han E, Choi AR, Ban TH, Chung BH, Yang CW, et al. Clinical impact of complement (C1q, C3d) binding De Novo donor-specific HLA antibody in kidney transplant recipients. PloS One. 2018;13(11):e0207434.

58. Malheiro J, Santos S, Tafulo S, Dias L, Martins LS, Fonseca I, et al. Detection of Complement-binding Donor-specific Antibodies, Not IgG-antibody Strength Nor C4d Status, at Antibody-mediated Rejection Diagnosis Is an Independent Predictor of Kidney Graft Failure. Transplantation. 2018 Nov;102(11):1943–54.

59. Schinstock CA, Dadhania DM, Everly MJ, Smith B, Gandhi M, Farkash E, et al. Factors at de novo donor-specific antibody initial detection associated with allograft loss: a multicenter study. Transpl Int. 2019;32(5):502–15.

60. Lee DR, Kim BC, Kim JP, Kim IG, Jeon MY. C3d-binding Donor-specific HLA Antibody Is Associated With a High Risk of Antibody-mediated Rejection and Graft Loss in Stable Kidney Transplant Recipients: A Single-center Cohort Study. Transplant Proc. 2018 Dec 1;50(10):3452–9.

61. Babu A, Khovanova N, Shaw O, Griffin S, Briggs D, Krishnan NS, et al. C3d-positive donor-specific antibodies have a role in pretransplant risk stratification of cross-match-positive HLA-incompatible renal transplantation: United Kingdom multicentre study. Transpl Int Off J Eur Soc Organ Transplant. 2020 Sep;33(9):1128–39.

62. Gautier Vargas G, Olagne J, Parissiadis A, Joly M, Cognard N, Perrin P, et al. Does a Useful Test Exist to Properly Evaluate the Pathogenicity of Donor-specific Antibodies? Lessons From a Comprehensive Analysis in a Well-studied Single-center Kidney Transplant Cohort. Transplantation. 2020 Oct;104(10):2148–57.

63. Zhang Q, Hickey M, Drogalis-Kim D, Zheng Y, Gjertson D, Cadeiras M, et al. Understanding the Correlation Between DSA, Complement Activation, and Antibody-Mediated Rejection in Heart Transplant Recipients. Transplantation. 2018 Oct;102(10):e431.

64. Cioni M, Nocera A, Tagliamacco A, Basso S, Innocente A, Fontana I, et al. Failure to remove de novo donor-specific HLA antibodies is influenced by antibody properties and identifies kidney recipients with late antibody-mediated rejection destined to graft loss – a retrospective study. Transpl Int. 2019;32(1):38–48.

65. Hayde N, Solomon S, Caglar E, Ge J, Qama E, Colovai A. C1q-binding DSA and allograft outcomes in pediatric kidney transplant recipients. Pediatr Transplant. 2021;25(2):e13885.

66. Pernin V, Beyze A, Szwarc I, Bec N, Salsac C, Perez-Garcia E, et al. Distribution of de novo Donor-Specific Antibody Subclasses Quantified by Mass Spectrometry: High IgG3 Proportion Is Associated With Antibody-Mediated Rejection Occurrence and Severity. Front Immunol. 2020;11:919.
